# Supplementary material for: Pleiotropy method reveals genetic overlap between orofacial clefts at multiple novel loci from GWAS of multi-ethnic trios
Source: PLoS Genet. 2021 Jul 9;17(7):e1009584. doi: 10.1371/journal.pgen.1009584 (PMC8270211; doi:10.1371/journal.pgen.1009584)
Supplement: S1 Appendix — It includes additional details on simulation experiments, power calculations, and PLACO implementation. (PDF) [file pgen.1009584.s001.pdf]

S1 Appendix

Empirical validation of sensitivity and specificity of PLACO.

Simulation setup: We simulated two bi-ethnic case-parent trio studies with a total of 2400 complete trios mimicking independent studies of CL/P and CP. We assumed, without loss of generality, that the two ethnic groups have equal sample sizes for a particular OFC subgroup, and considered situations where the OFC subgroups either have comparable/balanced (1:1) or unbalanced (3:1) or heavily unbalanced (7:1) sample sizes. For instance, the unbalanced (3:1) scenario simulates a total of 2400 trios of which 1800 are CL/P trios (900 in each ethnic group), and 600 are CP trios (300 in each ethnic group). Thus, the two studies with comparable (1:1) or unbalanced (3:1) or heavily unbalanced (7:1) sample sizes respectively have sample sizes ($n_{\mathrm{CL/P}}$, n_{CP}) = (1200,1200) or (1800,600) or (2100,300). Our choices of the unbalanced and the heavily unbalanced OFC subgroup sample sizes mimic the GENEVA and the POFC studies respectively (**S1 Table**).

We made the two ethnic groups distinct in two ways. First, we assumed that the OFC subgroup prevalence for each ethnic group is different. In particular, we used the cleft prevalence for Western Europe and South Asia[1]. CL/P prevalence for these two ethnic groups are 1.07/1000 and 1.30/1000, while those of CP are 0.59/1000 and 0.30/1000 respectively. Secondly, we assumed the minor allele frequency (MAF) of any given genetic variant is different between ethnic groups (as described next). Recall, the MAFs for the top SNPs from the regions of genetic overlap between CL/P and CP that we identified from the analysis of POFC + GENEVA data are mostly different across ethnic groups.

For the genetic data, we simulated 10 million independent bi-allelic genetic variants in Hardy-Weinberg equilibrium (HWE). We fixed the population-level MAF of one ethnic group at 10%; for the other ethnic group, we randomly selected the MAF between 7% and 13% using a continuous uniform distribution. We assumed the commonly-used additive genetic mode of inheritence, and used the gTDT conditional logistic model[2] as our outcome generative model for a given variant:

$$P\Big(Y_0 = 1 \Big| \{Y_0 + Y_1 + Y_2 + Y_3 = 1\}, \{G_0, G_1, G_2, G_3\}\Big) = \frac{e^{\beta G_0}}{e^{\beta G_0} + e^{\beta G_1} + e^{\beta G_2} + e^{\beta G_3}}$$

where $\beta = \log(\text{RR})$ is the genetic effect of the variant for a given OFC subgroup, Y_0 is the disease status of the child ($Y_0 = 1$ in a case-parent trio study), Y_l is the disease status of the l-th pseudo-control (takes value 0 for all pseudo-controls, $l = 1, 2, 3$), G_0 is the genotype of the child at the variant (coded additively as 0, 1, or 2 here), and G_l is the genotype of the l-th pseudo-control. Essentially, G_1, G_2 and G_3 are the possible genotypes at the bi-allelic variant that the child could have inherited from the parents. We emphasize that this generative model for our simulation experiments is distinct from the hierarchical model assumed by PLACO[3]. Since we need multiple independent replicates to assess type I error control and power at stringent error thresholds, we assumed the 10 million genetic variants are independent. Subsequently, we calculated estimated type I error (power) by averaging over the number of independent null (non-null) variants identified as having significant pleiotropic effect on both outcomes at a fixed significance level α (the choice of α is mentioned when presenting results).

Out of the 10 million genetic variants, we assumed 99% variants to be not associated with either of the two OFC subgroups (i.e., $\text{RR}_{\text{CL/P}} = 1$, $\text{RR}_{\text{CP}} = 1$), 0.5% variants to be associated with CP only (i.e., $\text{RR}_{\text{CL/P}} = 1$, $\text{RR}_{\text{CP}} \neq 1$), 0.4% variants to be associated with CL/P only (i.e., $\text{RR}_{\text{CL/P}} \neq 1$, $\text{RR}_{\text{CP}} = 1$), and 0.1% variants to be associated with both (i.e., $\text{RR}_{\text{CL/P}} \neq 1$, $\text{RR}_{\text{CP}} \neq 1$). Thus, our simulated dataset had 9.99 million null variants to estimate type I error and $10,000$ non-null variants to estimate statistical power. Note, we have purposefully simulated a very large number of null variants to enable meaningful type I error comparison between PLACO and other methods at stringent error thresholds typically used in GWAS. The different choices of $\text{RR}_{\text{CL/P}}$ and RR_{CP} for simulating our data will dictate the scenarios under which we evaluated type I error and power of PLACO in identifying variants having simultaneous association with CL/P and CP based on multi-ethnic case-parent trios.

Type I error comparisons: We considered two primary scenarios: we assumed either (I) fixed genetic effects for both OFC subgroups across variants, or (II) a distribution on the genetic effects of CL/P group and fixed genetic effects for CP. Specifically, for Scenario I we assumed 9.9 million null variants with $\{\text{RR}_{\text{CL/P}} = 1, \text{RR}_{\text{CP}} = 1\}$; $50,000$ null variants with $\{\text{RR}_{\text{CL/P}} = 1, \text{RR}_{\text{CP}} = 1.15\}$; and $40,000$ null variants with $\{\text{RR}_{\text{CL/P}} = 1.15, \text{RR}_{\text{CP}} = 1\}$. For Scenario II, we assumed 9.99 million null variants with $\log(\text{RR}_{\text{CL/P}})$ simulated from a normal

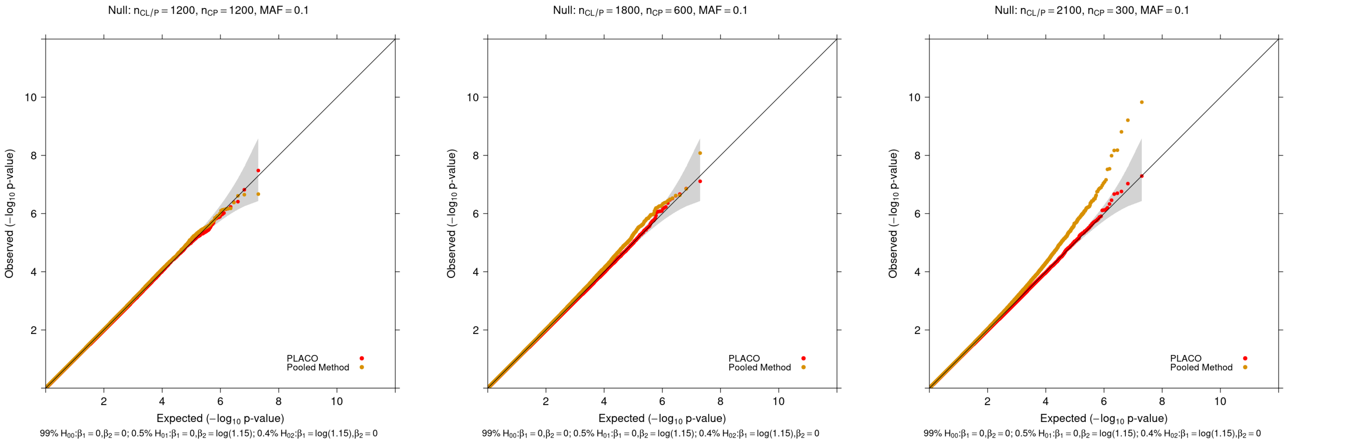

(a) MAF 10%; Balanced (1:1)

(b) MAF 10%; Unbalanced (3:1)

(c) MAF 10%; Heavily unbalanced (7:1)

Fig A1: [Same as S18 Fig] Scenario I: QQ plots for null data from two independent bi-ethnic case-parent trio studies of OFC subgroups assuming fixed genetic effects. Observed($-\log_{10}$p-values) are plotted on the y-axis and Expected($-\log_{10}$p-values) on the x-axis. Type I error performance of tests of simultaneous effect of a genetic variant on both outcomes is based on 9.99 million null variants with genetic effects that are either $\{RR_{CL/P} = 1, RR_{CP} = 1\}$ or $\{RR_{CL/P} = 1, RR_{CP} = 1.15\}$ or $\{RR_{CL/P} = 1.15, RR_{CP} = 1\}$. The gray shaded region represents a conservative 95% confidence interval for the expected distribution of p-values. P-values $\geq 10^{-12}$ are shown here.

distribution with mean 0, standard deviation 0.1, and fixed $RR_{CP} = 1$. The choice of this distribution for $\log(RR_{CL/P})$ is motivated by the distribution of effect sizes of common variants across many complex human traits[4]. Here all the variants are under the sub-null, where the RRs of variants for the CL/P group ranged from 1/1.7 to 1.7 (see **Table A1**). While Scenario I has bulk of the null variants under the global null (i.e., both genetic effects are null), Scenario II ensures that the bulk of null variants is under the sub-null where only one cleft group shows genetic association. Scenario I is simple and straightforward; Scenario II is more realistic.

Further, to evaluate sensitivity (if any) of PLACO's type I error control to varying MAF, we considered a simulation setting with everything the same as before except that the MAF of the variants for one ethnic group is fixed at 20% while that of the other ethnic group is allowed to vary more widely around 20% by simulating its MAF between 5% and 35% using a continuous uniform distribution. To avoid redundancy, we did this only for the more realistic Scenario II.

We compared PLACO with 'pooled method' GWAS analysis[5] previously used to identify risk variants common to both CL/P and CP. The pooled method combined the OFC subgroups together to form a single OFC group. When almost all of the null variants have no effect on either OFC subgroup (Scenario I), both PLACO and the pooled method appear to have controlled type

Table A1: Scenario II: Distribution of relative risks assumed in our simulated null data from two independent bi-ethnic case-parent trio studies of OFC subgroups assuming fixed genetic effects for one trait and random for the other. We simulated 9.99 million null variants with genetic effects $\{\log(\mathrm{RR}_{\mathrm{CL/P}}) \sim N(0, 0.1^2), \mathrm{RR}_{\mathrm{CP}} = 1\}$. Z-scores $Z_{\mathrm{CL/P}}$ and Z_{CP} are obtained using the gTDT on the trios.

$\mathrm{RR}_{\mathrm{CL/P}}$	$\mathrm{RR}_{\mathrm{CP}}$	No. of null variants	$Z_{\mathrm{CL/P}}$ min	$Z_{\mathrm{CL/P}}$ max	Z_{CP} min	Z_{CP} max
$[\frac{1}{1.7}, \frac{1}{1.6})$	1	15	-6.7	-4.2	-2.1	1.5
$[\frac{1}{1.6}, \frac{1}{1.5})$	1	230	-7.2	-1.4	-2.2	3.0
$[\frac{1}{1.5}, \frac{1}{1.4})$	1	3,702	-7.1	-0.5	-3.3	3.5
$[\frac{1}{1.4}, \frac{1}{1.3})$	1	32,608	-7.5	1.4	-4.0	5.0
$[\frac{1}{1.3}, \frac{1}{1.2})$	1	320,070	-7.3	8.0	-4.5	6.3
$[\frac{1}{1.2}, 1]$	1	4,841,998	-6.5	9.1	-4.8	6.3
(1,1.2]	1	4,490,581	-4.4	9.3	-4.7	6.3
(1.2, 1.3]	1	272,059	-1.6	8.1	-4.2	5.7
(1.3, 1.4]	1	35,305	-0.04	8.3	-4.4	4.2
(1.4, 1.5]	1	3,239	1.2	8.2	-3.6	3.9
(1.5, 1.6]	1	187	3.6	8.1	-3.0	3.2
(1.6, 1.7]	1	6	6.6	8.6	-1.5	1.6

(a,b] denotes the range $a < \mathrm{RR}_{\mathrm{CL/P}} \leq b$. Similarly, [a,b) denotes $a \leq \mathrm{RR}_{\mathrm{CL/P}} < b$.

I error for balanced sample sizes of the OFC subgroups (**Figure A1 (a)**). As the OFC subgroups become more and more skewed in terms of sample size (as seen for our GENEVA and POFC studies), the pooled method shows inflated type I error while PLACO maintains appropriate type I error rate even at stringent error levels (**Figure A1 (b)-(c)**). This shows that the 'pooled method' does not necessarily capture only shared signals[6]; it may show spurious signals if sample sizes are widely different for the subgroups (e.g., CL/P group is almost always much larger than CP group).

When a large proportion of null variants have genetic effect on one OFC subgroup only with the genetic effect randomly ranging from being weak to strong (Scenario II), the pooled method shows hugely inflated type I error rate. Even in this sub-null scenario consisting of many variants with large RRs for one subgroup only (**Table A1**), we find PLACO maintains type I error at stringent levels regardless of how skewed the sample sizes are between the two OFC subgroups (**Figure A2 (a)-(c)**). All the variants on the PLACO QQ plots appear to be contained within the 95% confidence interval for the expected distribution of p-values. These observations hold true for varying MAFs irrespective of how widely different the MAFs are between the two ethnic groups (**Figure A2 (d)-(f)**).

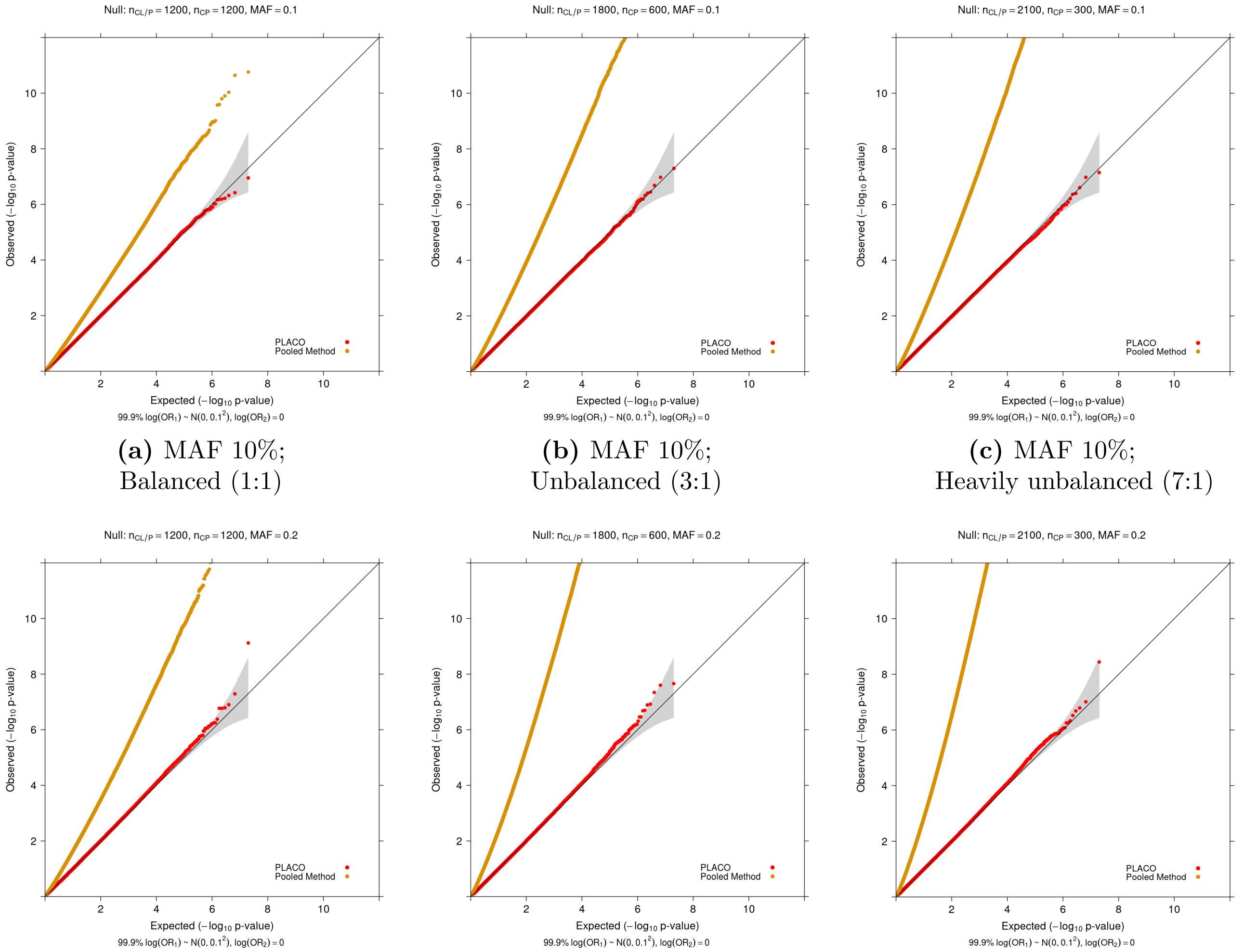

(a) MAF 10%; Balanced (1:1)

(b) MAF 10%; Unbalanced (3:1)

(c) MAF 10%; Heavily unbalanced (7:1)

(d) MAF 20%; Balanced (1:1)

(e) MAF 20%; Unbalanced (3:1)

(f) MAF 20%; Heavily unbalanced (7:1)

Fig A2: [Same as S19 Fig] Scenario II: QQ plots for null data from two independent bi-ethnic case-parent trio studies of OFC subgroups assuming fixed genetic effects for one trait and random for the other. Observed($-\log_{10}$p-values) are plotted on the y-axis and Expected($-\log_{10}$p-values) on the x-axis. Type I error performance of tests of simultaneous effect of a genetic variant on both outcomes is based on 9.99 million null variants with genetic effects $\{\log(\text{RR}_{\text{CL/P}}) \sim N(0, 0.1^2), \text{RR}_{\text{CP}} = 1\}$. The gray shaded region represents a conservative 95% confidence interval for the expected distribution of p-values. P-values $\geq 10^{-12}$ are shown here.

Comparisons with meta-analysis methods: Apart from the pooled method, we included other potential methods such as meta-analysis techniques in our type I error comparisons. Theoretically, the pooled method and any meta-analysis technique test the same global null hypothesis, which is not exactly the null hypothesis to test when the goal is to identify common genetic basis of two disease subgroups or two disorders. One can employ a fixed effect inverse-variance weighted meta-analysis, which is equivalent to the pooled method as has been shown in statistical literature before[7]. Other meta-analysis techniques that ignore directions of effects involve combination of

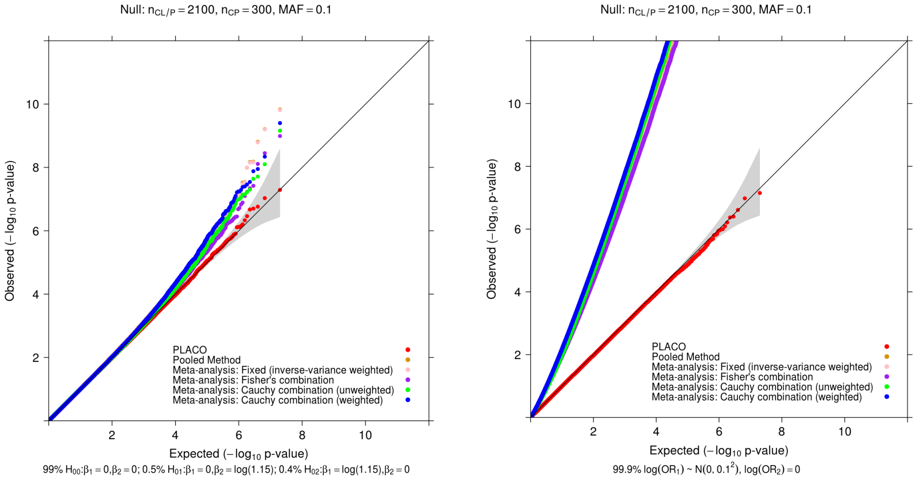

(a) Scenario I **(b)** Scenario II

Fig A3: QQ plots for null data from two independent bi-ethnic case-parent trio studies of OFC subgroups comparing PLACO and the pooled method against different meta-analysis methods. Observed($-\log_{10}$p-values) are plotted on the y-axis and Expected($-\log_{10}$p-values) on the x-axis. Type I error performance of tests of simultaneous effect of a genetic variant on both outcomes is based on 9.99 million null variants. Under Scenario I where fixed genetic effects are assumed for both traits, 9.9 million variants have genetic effects $\{\mathrm{RR_{CL/P}} = 1, \mathrm{RR_{CP}} = 1\}$, $50,000$ variants satisfy $\{\mathrm{RR_{CL/P}} = 1, \mathrm{RR_{CP}} = 1.15\}$, and $40,000$ variants satisfy $\{\mathrm{RR_{CL/P}} = 1.15, \mathrm{RR_{CP}} = 1\}$. Under Scenario II where fixed genetic effect is assumed for one trait and random for the other, the 9.99 million null variants have genetic effects $\{\log(\mathrm{RR_{CL/P}}) \sim N(0, 0.1^2), \mathrm{RR_{CP}} = 1\}$. The gray shaded region represents a conservative 95% confidence interval for the expected distribution of p-values. P-values $\geq 10^{-12}$ are shown here.

p-values from the cleft subgroups: Fisher's combination[8], and Cauchy combination[9]. We used the `CCT` function from R package `STAAR`[10] to implement the Cauchy combination method in two ways: unweighted, and sample size weighted. As expected, these meta-analysis methods and the pooled method have comparable performance: all show lack of type I error control in most scenarios (**Figure A3**). Consequently, we did not further consider these meta-analysis methods for power comparison.

These type I error simulation experiments show that the 'pooled method' (and equivalently the different meta-analysis techniques) is prone to exhibiting spurious signals if genetic effects exist in one OFC subgroup but not the other. On the other hand, we observe the robustness of PLACO's type I error control to sample size differences between OFC subgroups; moderately strong subgroup-specific effects; and small to large MAF differences between ethnic groups.

Power comparisons: For the $10,000$ non-null variants in our simulated dataset used to estimate power, we considered different choices of the two relative risks $\mathrm{RR_{CL/P}}$ and $\mathrm{RR_{CP}}$ to reflect genetic effects of varying directions and/or magnitudes. For benchmarking, we compared power

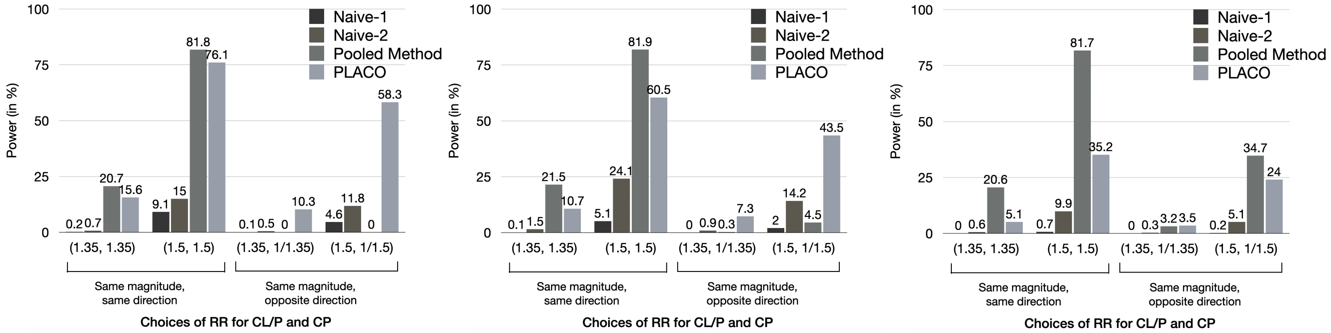

(a) MAF 10%; Balanced (1:1)

(b) MAF 10%; Unbalanced (3:1)

(c) MAF 10%; Heavily unbalanced (7:1)

Fig A4: [Same as S20 Fig] Power of PLACO, pooled method, and naive approaches at genome-wide significance level (5×10^{-8}) for varying genetic effects of the two independent bi-ethnic case-parent trio studies of OFC subgroups. The first naive approach ('Naive-1') declares pleiotropic association when $p_{\mathrm{CL/P}} < 5 \times 10^{-8}$ and $p_{\mathrm{CP}} < 10^{-5}$, while the second naive approach ('Naive-2') uses a more liberal criterion $p_{\mathrm{CL/P}} < 5 \times 10^{-8}$ and $p_{\mathrm{CP}} < 10^{-3}$. Note, unlike PLACO, the pooled method lacks type I error control in most scenarios of sample size and/or MAF imbalance, and hence its power should be interpreted with caution.

of PLACO against the pooled method (even though pooled method showed inflated type I error in most of our simulated scenarios). We also included the naive approach of declaring genetic overlap when a variant reaches genome-wide significance for the OFC subgroup with a larger sample size (in our case, CL/P) and reaches a more liberal significance threshold for the other. We used two such naive approaches: one based on criterion $p_{\mathrm{CL/P}} < 5 \times 10^{-8}$, $p_{\mathrm{CP}} < 10^{-5}$ and the other $p_{\mathrm{CL/P}} < 5 \times 10^{-8}$, $p_{\mathrm{CP}} < 10^{-3}$ ('Naive-1' and 'Naive-2' respectively in our figures). Regardless of the magnitude and directions of simultaneous association and the sample size differences between OFC subgroups, PLACO shows dramatically improved statistical power to detect common genetic basis compared to the naive approaches (**Figure A4**). Pooled method is slightly more powerful than PLACO in identifying shared risk variants (i.e., variants with genetic effects on the two OFC subgroups in the same direction); however, it especially lacks power to detect variants that increase risk for one OFC subgroup while decreasing risk for the other. Note that for the hugely unbalanced (7:1) sample size scenario, the pooled method appears to be more powerful than PLACO for opposite genetic effects; this behavior is explained by the fact that pooled method is strongly influenced (biased away from the null) by sample size differences between groups, and its type I error control can be particularly bad in this scenario (**Figures A1(c)**, **A2(c)** and **A2(f)**).

Power of PLACO analysis stratified by race/ethnicity.

In this paper, we have explored if locus-specific effects at regions of genetic overlap we identified between CL/P & CP vary by racial/ethnic group. To estimate the power of such an analysis, we performed some simulation experiments mimicking the lead SNPs of 2 loci from **Table 1**: 1p36.13 (*PAX7*) and 1q32.2 (*IRF6*). We simulated 2,153 CL/P case-parent trios and 569 CP case-parent trios. For each cleft subgroup, we simulated 2 racial/ethnic groups: Group1 (Group2) closely mimicking the European (Asian) ancestry trios in our study. Note, we have trios with Latin American ancestry in our study as well (albeit small sample size); here we simulated only 2 groups for simplicity. **Table A2** provides details of all the parameter values chosen for these simulations. The two ethnic groups are made distinct in three ways: OFC subgroup prevalence, the causal effect size (or relative risk) and the allele frequency of a variant for each ethnic group are different. The model used to generate the simulated data is the same as described before. For each chosen SNP, we simulated 10 million replicates of which 9.99 million are null and $10,000$ are non-null (see **Table A2** for the magnitude of effects chosen in each subgroup). We generated both null and non-null variants, mimicking genome-wide scale, so as to be able to estimate the parameters needed to calculate PLACO p-value. The $10,000$ non-null replicates were then used to estimate statistical power.

We found that PLACO has 71% (11%) power to detect simultaneous association of SNP rs1339063 (*PAX7*) with CL/P and CP in the European (Asian) ancestry at a liberal significance threshold of 10^{-4}. We are using a liberal threshold for these stratified analyses since we looked at ethnicity-specific results only at the regions of genetic overlap PLACO identified between CL/P & CP using multi-ethnic trios in the primary analysis. Similarly, PLACO's power is, respectively, 21% and 98% for SNP rs72741048 (*IRF6*) in our European and Asian groups. This differential power for the two racial/ethnic groups at these two SNPs in our simulated data is also reflected in our study of GENEVA and POFC trios. In our study, we found signals of genetic overlap at the *PAX7* locus is driven by the European subjects (**S7 Fig C-E**) while the *IRF6* locus seems to draw upon evidence from the Asian subjects (**S3 Fig C-E**).

Table A2: Parameters used in the simulation experiments to determine power of our race/ethnicity stratified analysis using PLACO. Simulations are mimicking the lead SNPs of loci 1p36.13 (*PAX7*) and 1q32.2 (*IRF6*) from Table 1. Ethnic Group1 (Group2) is modeled after the trios from European (Asian) ancestry in our study. The chosen relative risk (RR) parameters are based on the RR estimates from the gTDT analysis of each cleft subgroup within each ethnic group. The effect allele frequencies are based on the estimates from the parents in our study. Sample sizes are exactly the same as in our study. For the prevalence parameter, we used the cleft prevalence for Western Europe and South Asia by Mossey and Modell[1].

	CL/P		CP	
	Group1	Group2	Group1	Group2
No. of trios	978	1175	296	273
Prevalence	1.07/1000	1.30/1000	0.59/1000	0.30/1000

Locus (gene)	rsID	CL/P RR		CP RR		Effect allele freq.	
		Group1	Group2	Group1	Group2	Group1	Group2
1p36.13 (*PAX7*)	rs1339063	1.40	1.20	0.77	0.76	0.45	0.18
1q32.2 (*IRF6*)	rs72741048	0.76	0.63	1.24	1.44	0.21	0.43

Using PLACO program on GWAS summary statistics.

We followed the instructions and the toy example demonstration in the PLACO v0.1.1 manual (`https://github.com/RayDebashree/PLACO`) to conduct our genetic overlap analyses of different pairwise combination of cleft subtypes. There are two steps to implementing PLACO in R[11] on genome-wide summary statistics:

1. Organize the summary statistics and obtain variance parameter estimates (done only once for a GWAS)

   1a. If summary statistics (i.e., effect estimates, their standard errors and p-values, or Z-scores and p-values) are not available, then obtain them using a suitable method. Note, p-values, such as those from allelic TDT type approaches, alone cannot be used. Also, the effect allele needs to be harmonized for the two disease subgroups.

   1b. If Z-scores are available then arrange them in a matrix with the 2 disease subgroups along columns, and the SNPs along rows. If only log(RR) estimates and their standard errors are available, then create the matrix of Z-scores using the formula $Z = \frac{\log(\text{RR})}{\text{se}(\log(\text{RR}))}$. Remove SNPs for which the squared Z-score exceeds 80 for one or both subgroups. Similarly, arrange the corresponding p-values in a separate matrix.

   1c. Apply the `var.placo` function from the PLACO R program. This function requires a choice for parameter `p.threshold`, a preliminary p-value threshold used to select a set of possibly null SNPs genome-wide to estimate the variance parameters needed

to compute the PLACO p-value for a given SNP[3]. Ray and Chatterjee[3] did not find PLACO to be sensitive to the choice of this `p.threshold` parameter, so we used the default value of 10^{-4}. Note, this is *not* the significance threshold used to declare significant results from PLACO.

2. Apply `placo` function one SNP at a time.

   Note, this function inputs the Z-scores of two disease subgroups for a chosen SNP and the estimated variance parameter from Step 1c above to output the value of PLACO test-statistic and its p-value. This step does not involve any parameter choice.

As outlined in the manuscript, we used the gTDT from R `trio` package[12] on our trio data to obtain the GWAS summary statistics. If there is a large number of dyads along with trios, one may obtain the summary statistics using the generalized disequilibrium test (GDT)[13]. PLACO may also be used on summary statistics from a log-linear model[14;15]. Since there are multiple RR estimates from a log-linear model on trios, one needs to decide which RR estimate to apply PLACO on. For instance, one can test if CL/P and CP both have non-zero single dose child allele effects. If one is interested in the double dose child allele effects as well, then one needs to apply PLACO separately on the Z-scores obtained from double dose child allele effect estimates from the same log-linear model. PLACO cannot automatically combine single and double dose effect estimates to provide a single answer. Other models may also be used; the user needs to judge how to interpret the findings and if it violates any basic modeling assumption underlying PLACO.

References

[1] Mossey, P. and Modell, B. Epidemiology of oral clefts 2012: an international perspective. In *Cleft lip and palate*, volume 16, pages 1–18. Karger Publishers, 2012.

[2] Schwender, H., Taub, M. A., Beaty, T. H., Marazita, M. L., and Ruczinski, I. Rapid testing of SNPs and gene-environment interactions in case-parent trio data based on exact analytic parameter estimation. *Biometrics*, 68(3):766–773, 2012.

[3] Ray, D. and Chatterjee, N. A powerful method for pleiotropic analysis under composite null

hypothesis identifies novel shared loci between type 2 diabetes and prostate cancer. *PLoS Genet*, 16(12):e1009218, 2020.

[4] Zhang, Y., Qi, G., Park, J.-H., and Chatterjee, N. Estimation of complex effect-size distributions using summary-level statistics from genome-wide association studies across 32 complex traits. *Nat Genet*, 50(9):1318–1326, 2018.

[5] Leslie, E. J., Carlson, J. C., Shaffer, J. R., Butali, A., Buxó, C. J., Castilla, E. E., Christensen, K., Deleyiannis, F. W., Field, L. L., Hecht, J. T., et al. Genome-wide meta-analyses of nonsyndromic orofacial clefts identify novel associations between *FOXE1* and all orofacial clefts, and *TP63* and cleft lip with or without cleft palate. *Hum Genet*, 136(3):275–286, 2017.

[6] Carlson, J. C. *Methods for family-based designs in genetic epidemiology studies.* PhD thesis, University of Pittsburgh, 2017.

[7] Lin, D. and Zeng, D. Meta-analysis of genome-wide association studies: no efficiency gain in using individual participant data. *Genet Epidemiol*, 34(1):60–66, 2010.

[8] Ray, D., Pankow, J. S., and Basu, S. USAT: A unified score-based association test for multiple phenotype-genotype analysis. *Genet Epidemiol*, 40(1):20–34, 2016.

[9] Liu, Y. and Xie, J. Cauchy combination test: a powerful test with analytic p-value calculation under arbitrary dependency structures. *J Am Stat Assoc*, 115(529):393–402, 2020.

[10] Li, X., Li, Z., Zhou, H., Gaynor, S. M., Liu, Y., Chen, H., Sun, R., Dey, R., Arnett, D. K., Aslibekyan, S., et al. Dynamic incorporation of multiple in silico functional annotations empowers rare variant association analysis of large whole-genome sequencing studies at scale. *Nat Genet*, 52(9):969–983, 2020.

[11] R Core Team. *R: A Language and Environment for Statistical Computing.* R Foundation for Statistical Computing, Vienna, Austria, 2018. URL `https://www.R-project.org/`.

[12] Schwender, H., Li, Q., Neumann, C., Taub, M. A., Younkin, S. G., Berger, P., Scharpf, R. B., Beaty, T. H., and Ruczinski, I. Detecting disease variants in case-parent trio studies using the bioconductor software package `trio`. *Genet Epidemiol*, 38(6):516–522, 2014.

[13] Chen, W.-M., Manichaikul, A., and Rich, S. S. A generalized family-based association test for dichotomous traits. *Am J Hum Genet*, 85(3):364–376, 2009.

[14] Weinberg, C., Wilcox, A., and Lie, R. A log-linear approach to case-parent–triad data: assessing effects of disease genes that act either directly or through maternal effects and that may be subject to parental imprinting. *Am J Hum Genet*, 62(4):969–978, 1998.

[15] Weinberg, C. Allowing for missing parents in genetic studies of case-parent triads. *Am J Hum Genet*, 64(4):1186–1193, 1999.
