# Supplementary material for: Pleiotropy method reveals genetic overlap between orofacial clefts at multiple novel loci from GWAS of multi-ethnic trios
Source: PLoS Genet. 2021 Jul 9;17(7):e1009584. doi: 10.1371/journal.pgen.1009584 (PMC8270211; doi:10.1371/journal.pgen.1009584)
Supplement: S1 Fig — (PDF) [file pgen.1009584.s002.pdf]

Ray, D. et al. (2021) *Pleiotropy method reveals genetic overlap between orofacial clefts at multiple novel loci from GWAS of multi-ethnic trios.* PLoS Genetics, 17:e1009584, DOI: 10.1371/journal.pgen.1009584

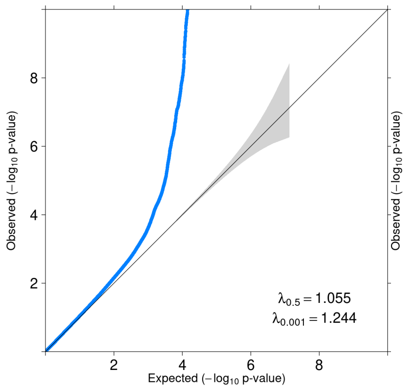

(A) CL/P (All ethnicities)

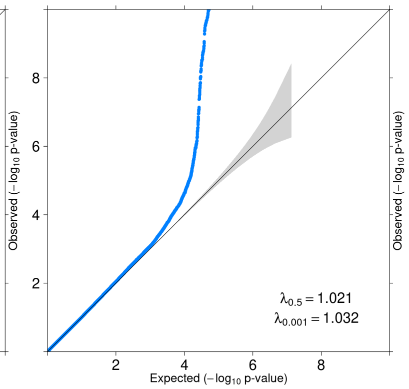

(B) CL/P (European)

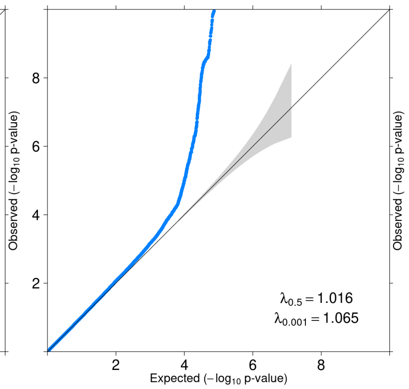

(C) CL/P (Asian)

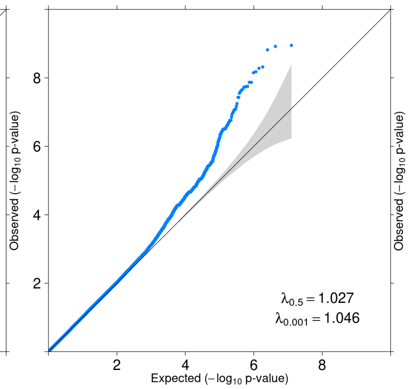

(D) CL/P (Latinx)

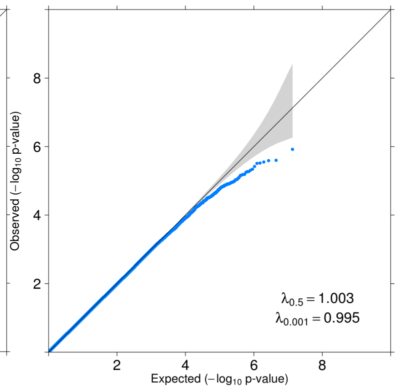

(E) CL/P (All ethnicities) - SNP×Sex analysis

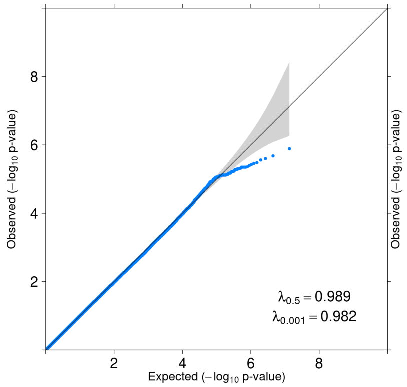

(F) CP (All ethnicities)

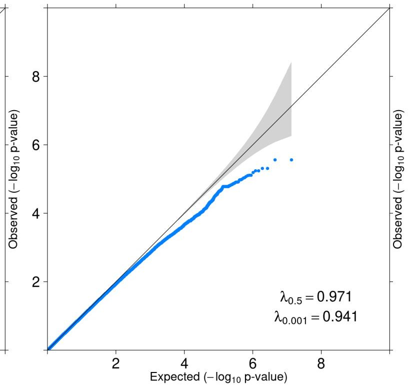

(G) CP (European)

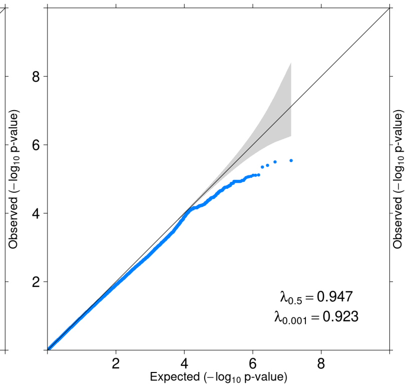

(H) CP (Asian)

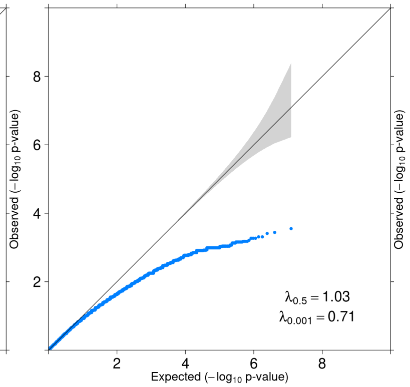

(I) CP (Latinx)

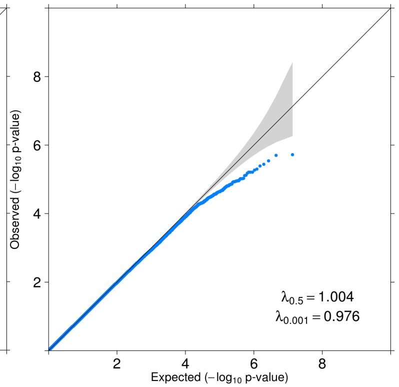

(J) CP (All ethnicities) - SNP×Sex analysis

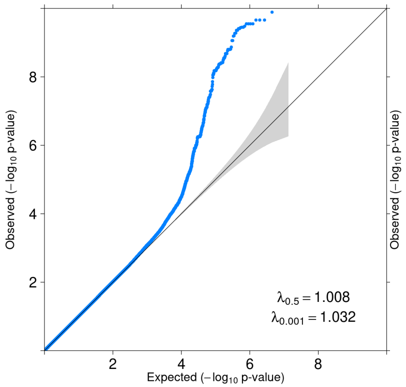

(K) CL (All ethnicities)

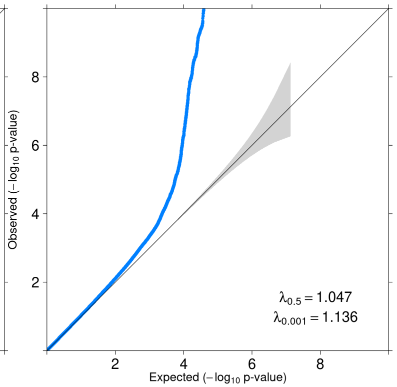

(L) CLP (All ethnicities)

S1 Fig: QQ plots of the gTDT summary statistics used for different genetic overlap analysis using PLACO in this manuscript.
