# Supplementary material for: Pleiotropy method reveals genetic overlap between orofacial clefts at multiple novel loci from GWAS of multi-ethnic trios
Source: PLoS Genet. 2021 Jul 9;17(7):e1009584. doi: 10.1371/journal.pgen.1009584 (PMC8270211; doi:10.1371/journal.pgen.1009584)
Supplement: S2 Fig — (PDF) [file pgen.1009584.s003.pdf]

Ray, D. et al. (2021) *Pleiotropy method reveals genetic overlap between orofacial clefts at multiple novel loci from GWAS of multi-ethnic trios.* PLoS Genetics, 17:e1009584, DOI: 10.1371/journal.pgen.1009584

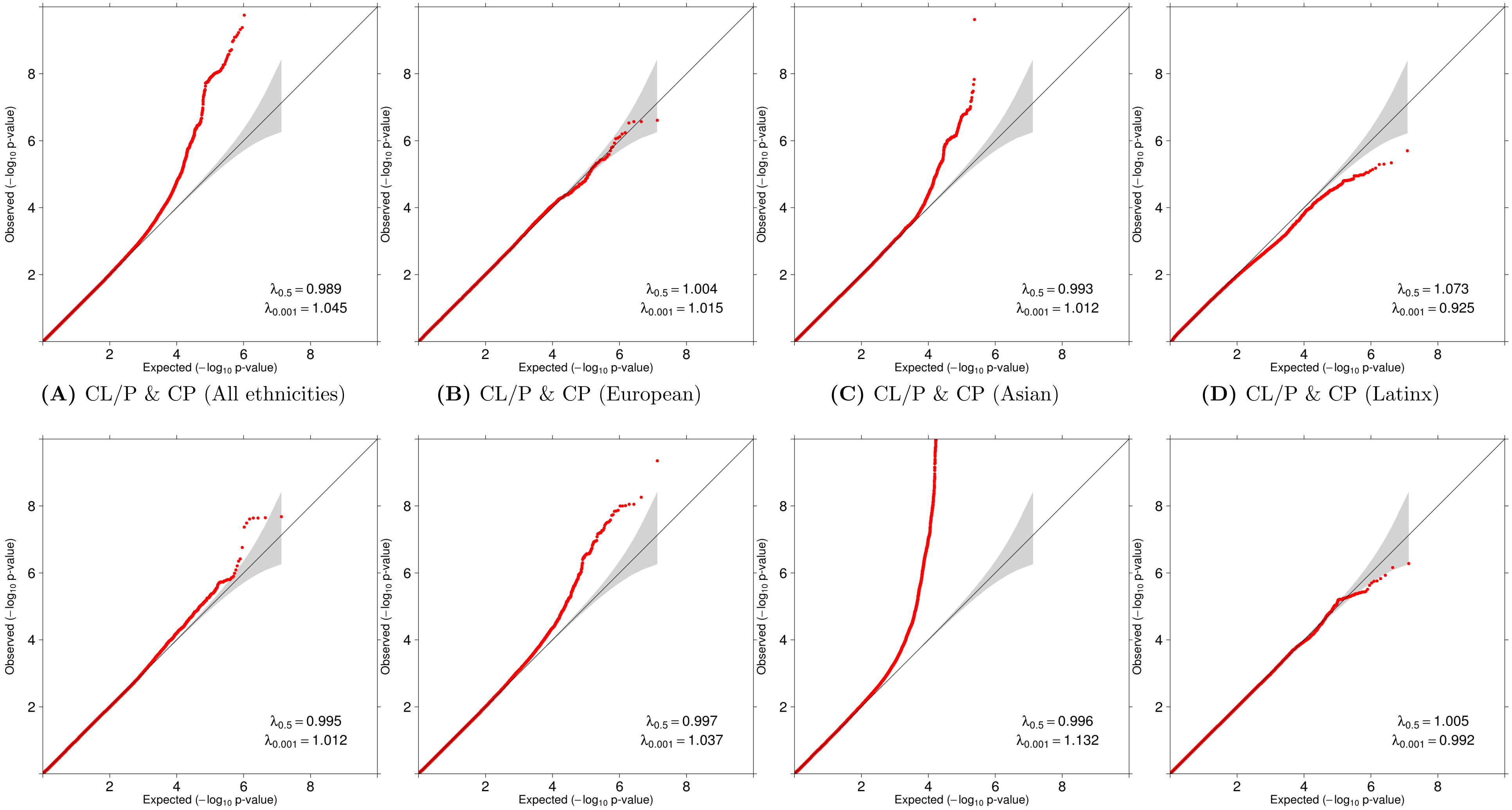

(A) CL/P & CP (All ethnicities) **(B)** CL/P & CP (European) **(C)** CL/P & CP (Asian) **(D)** CL/P & CP (Latinx)

(E) CL & CP (All ethnicities) **(F)** CLP & CP (All ethnicities) **(G)** CL & CLP (All ethnicities) **(H)** CL/P & CP (All ethnicities) - SNP×Sex analysis

S2 Fig: QQ plots from the different PLACO analyses, including stratified analyses, conducted in this manuscript.
