# Supplementary material for: Pleiotropy method reveals genetic overlap between orofacial clefts at multiple novel loci from GWAS of multi-ethnic trios
Source: PLoS Genet. 2021 Jul 9;17(7):e1009584. doi: 10.1371/journal.pgen.1009584 (PMC8270211; doi:10.1371/journal.pgen.1009584)
Supplement: S6 Fig — Index SNP here is the lead (most significant) SNP at each locus. SNPs with opposite genetic effects for 2 OFC subgroups are colored in golden yellow while those with shared effects are in dark green. The directions of genetic effects are determined from the relative risk (RR) estimates for each subgroup as provided by the gTDT method. RR estimates and the corresponding 95% confidence intervals for the SNPs above the dashed red horizontal line are portrayed in Fig 2. (PDF) [file pgen.1009584.s007.pdf]

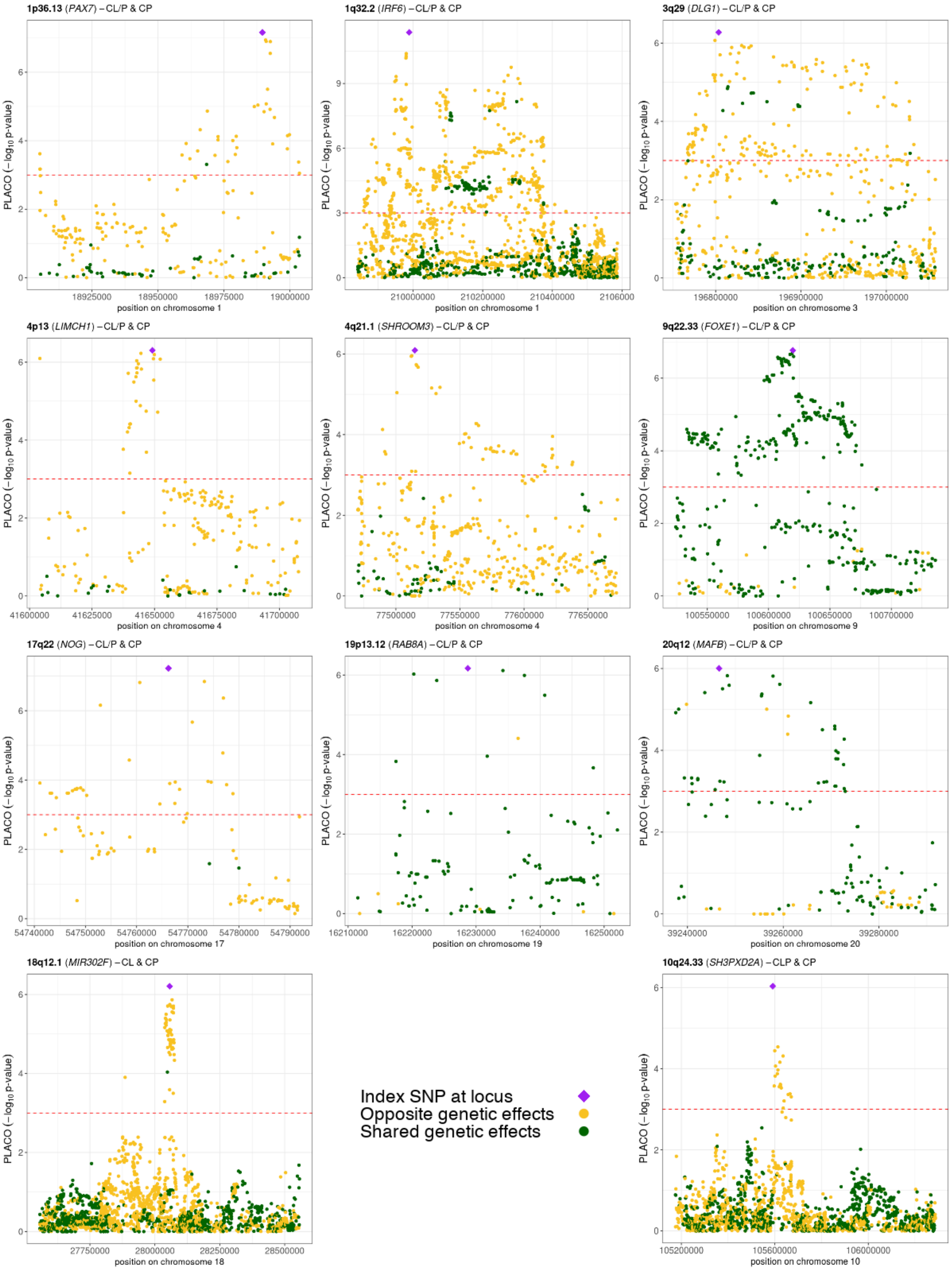

S6 Fig: Regional association plots of PLACO p-values, annotated by directions of effect sizes, for variants in the 9 loci showing statistical evidence of genetic overlap between CL/P & CP, along with 2 additional loci of genetic overlap between component OFC subtypes. Index SNP here is the lead (most significant) SNP at each locus. SNPs with opposite genetic effects for 2 OFC subgroups are colored in golden yellow while those with shared effects are in dark green. The directions of genetic effects are determined from the relative risk (RR) estimates for each subgroup as provided by the gTDT method. RR estimates and the corresponding 95% confidence intervals for the SNPs above the dashed red horizontal line are portrayed in Fig 2.
