# Supplementary material for: Pleiotropy method reveals genetic overlap between orofacial clefts at multiple novel loci from GWAS of multi-ethnic trios
Source: PLoS Genet. 2021 Jul 9;17(7):e1009584. doi: 10.1371/journal.pgen.1009584 (PMC8270211; doi:10.1371/journal.pgen.1009584)
Supplement: S11 Fig — LocusZoom plots focus on PLACO analysis of (A) CL/P & CP, (C) CL/P & CP in Asian ancestry, (D) CL/P & CP in European ancestry, (E) CL/P & CP in Latin American ancestry, (F) CL & CP, (G) CLP & CP, (H) CL & CLP. The blue or purple diamond represents the most strongly associated SNP in the region showing evidence of genetic overlap. For stratified analyses across racial/ethnic groups, the colors of the SNPs represent their LD with the most strongly associated SNP, as shown in the color legend. For combined multi-ethnic analyses, there is no unique LD between SNPs and hence no color has been used. Panel (B) shows relative risk estimates and their 95% confidence intervals as obtained from the gTDT analyses. (PDF) [file pgen.1009584.s012.pdf]

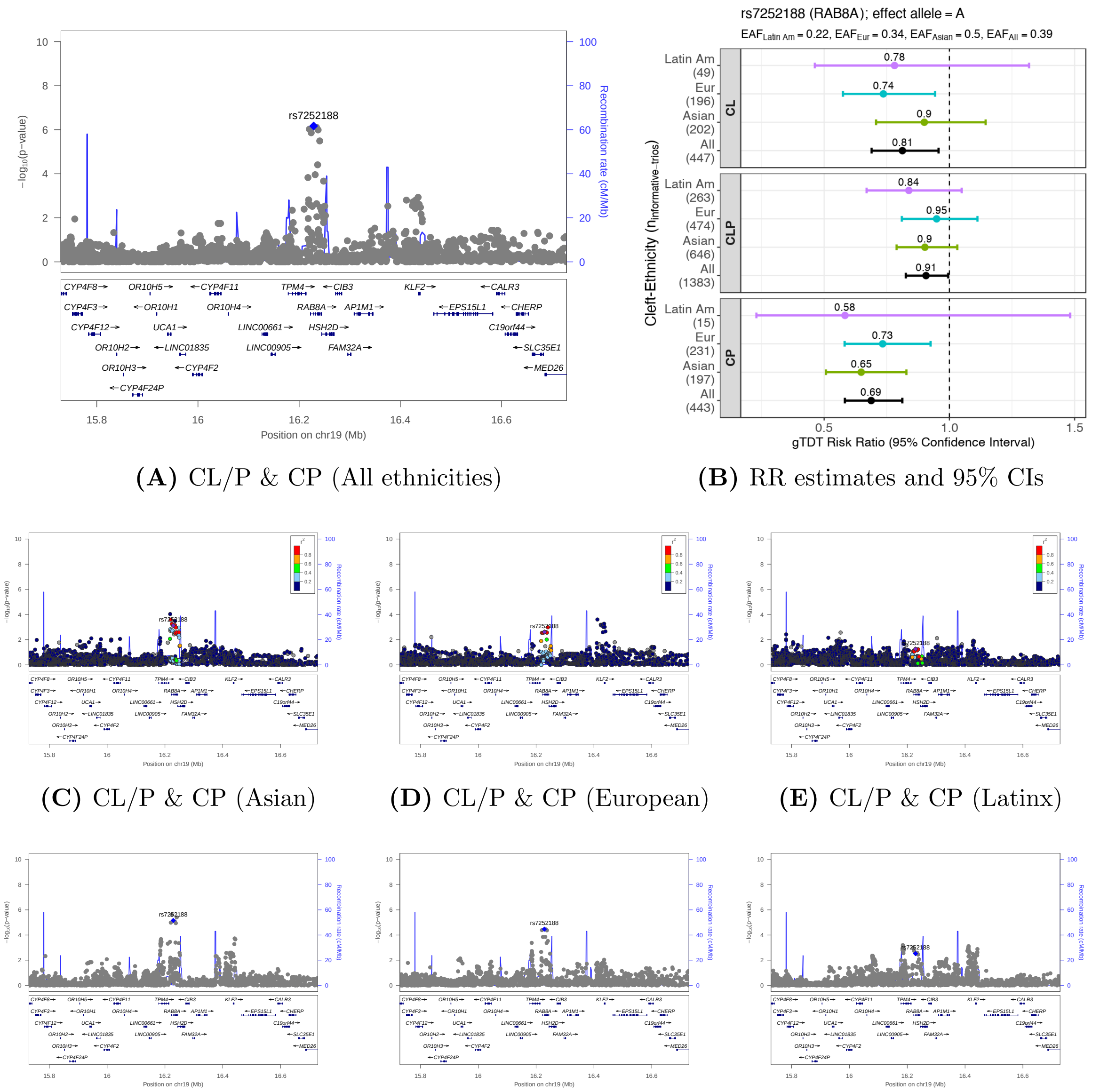

(A) CL/P & CP (All ethnicities) **(B)** RR estimates and 95% CIs

(C) CL/P & CP (Asian) **(D)** CL/P & CP (European) **(E)** CL/P & CP (Latinx)

(F) CL & CP (All ethnicities) **(G)** CLP & CP (All ethnicities) **(H)** CL & CLP (All ethnicities)

S11 Fig: Regional association plots for 19p13.12 (*RAB8A*) identified as a region of genetic overlap between CL/P & CP. LocusZoom plots focus on PLACO analysis of **(A)** CL/P & CP, **(C)** CL/P & CP in Asian ancestry, **(D)** CL/P & CP in European ancestry, **(E)** CL/P & CP in Latin American ancestry, **(F)** CL & CP, **(G)** CLP & CP, **(H)** CL & CLP. The blue or purple diamond represents the most strongly associated SNP in the region showing evidence of genetic overlap. For stratified analyses across racial/ethnic groups, the colors of the SNPs represent their LD with the most strongly associated SNP, as shown in the color legend. For combined multi-ethnic analyses, there is no unique LD between SNPs and hence no color has been used. Panel **(B)** shows relative risk estimates and their 95% confidence intervals as obtained from the gTDT analyses.
