# Supplementary material for: Pleiotropy method reveals genetic overlap between orofacial clefts at multiple novel loci from GWAS of multi-ethnic trios
Source: PLoS Genet. 2021 Jul 9;17(7):e1009584. doi: 10.1371/journal.pgen.1009584 (PMC8270211; doi:10.1371/journal.pgen.1009584)
Supplement: S12 Fig — LocusZoom plots focus on PLACO analysis of (A) CL/P & CP, (C) CL & CP, (D) CLP & CP, (E) CL & CLP. The blue diamond represents the most strongly associated SNP in the region showing evidence of genetic overlap. For multi-ethnic analyses, there is no unique LD between SNPs and hence no color has been used to represent strength of LD. Panel (B) shows relative risk estimates and their 95% confidence intervals as obtained from the gTDT analyses. (PDF) [file pgen.1009584.s013.pdf]

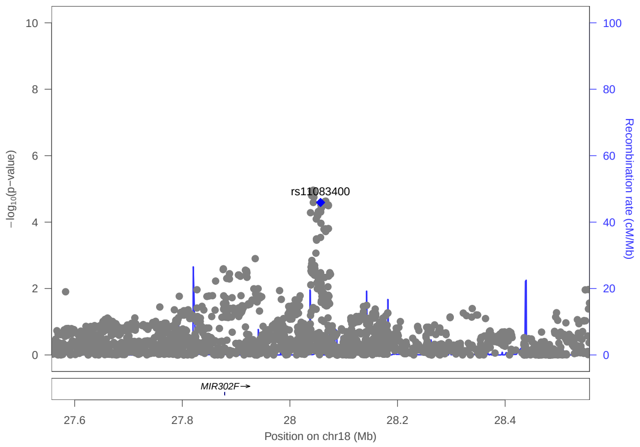

(A) CL/P & CP (All ethnicities)

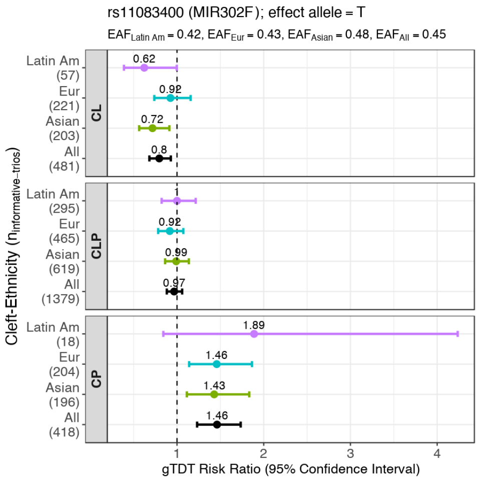

(B) RR estimates and 95% CIs

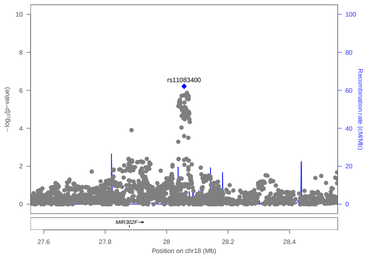

(C) CL & CP (All ethnicities)

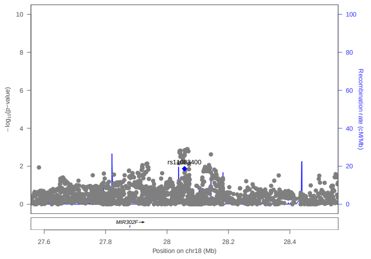

(D) CLP & CP (All ethnicities)

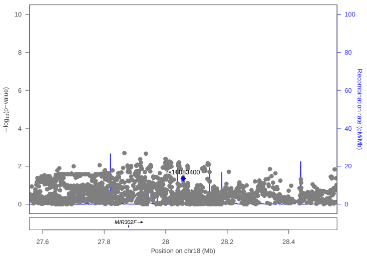

(E) CL & CLP (All ethnicities)

S12 Fig: Regional association plots for 18q12.1 (*MIR302F*) identified as a region of genetic overlap between CL & CP. LocusZoom plots focus on PLACO analysis of **(A)** CL/P & CP, **(C)** CL & CP, **(D)** CLP & CP, **(E)** CL & CLP. The blue diamond represents the most strongly associated SNP in the region showing evidence of genetic overlap. For multi-ethnic analyses, there is no unique LD between SNPs and hence no color has been used to represent strength of LD. Panel **(B)** shows relative risk estimates and their 95% confidence intervals as obtained from the gTDT analyses.
