# Supplementary material for: Pleiotropy method reveals genetic overlap between orofacial clefts at multiple novel loci from GWAS of multi-ethnic trios
Source: PLoS Genet. 2021 Jul 9;17(7):e1009584. doi: 10.1371/journal.pgen.1009584 (PMC8270211; doi:10.1371/journal.pgen.1009584)
Supplement: S14 Fig — P-values from our SNP × Sex analyses are plotted. The blue diamond represents the most strongly associated SNP in the region of genetic overlap. For multi-ethnic analyses, there is no unique LD between SNPs and hence no color has been used to represent strength of LD. (PDF) [file pgen.1009584.s015.pdf]

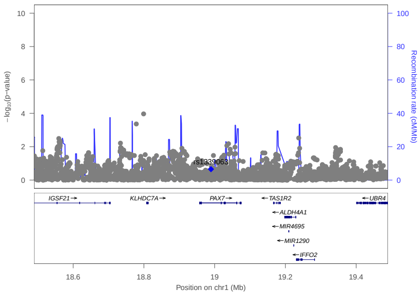

(A) *PAX7*

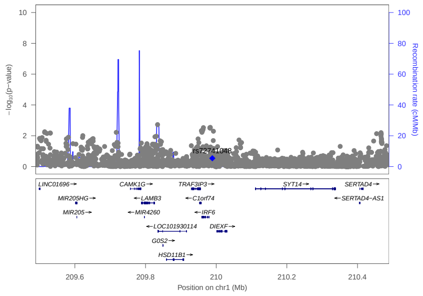

(B) *IRF6*

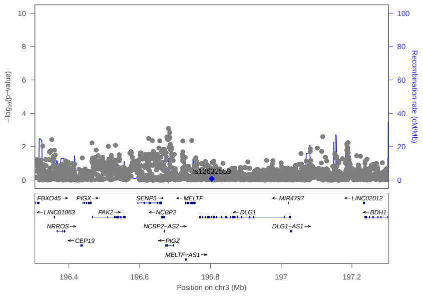

(C) *DLG1*

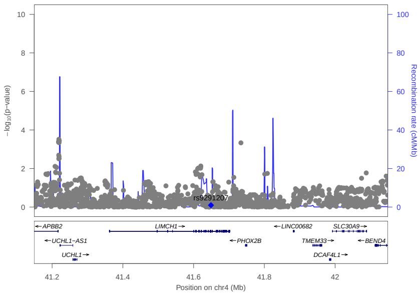

(D) *LIMCH1*

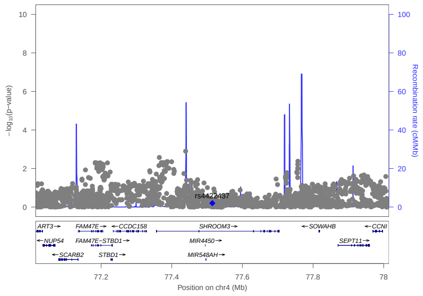

(E) *SHROOM3*

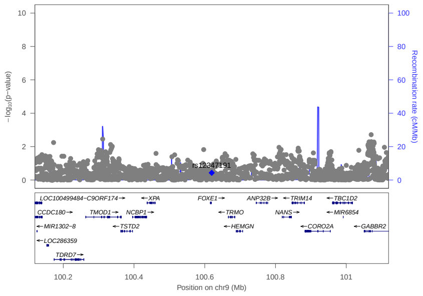

(F) *FOXE1*

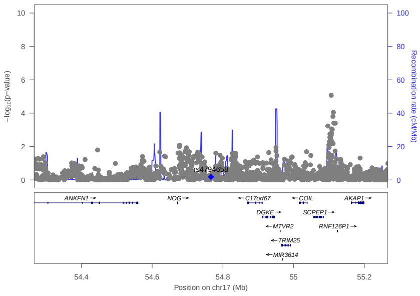

(G) *NOG*

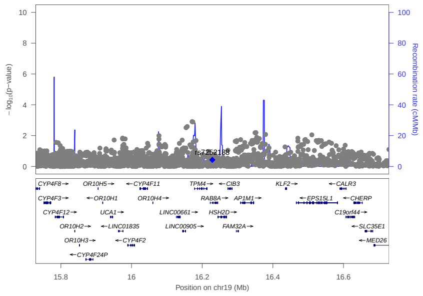

(H) *RAB8A*

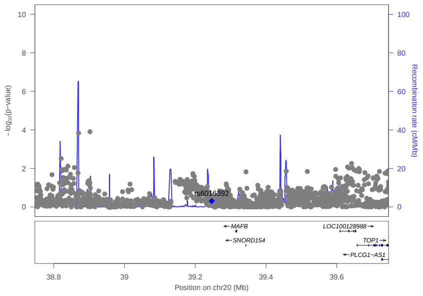

(I) *MAFB*

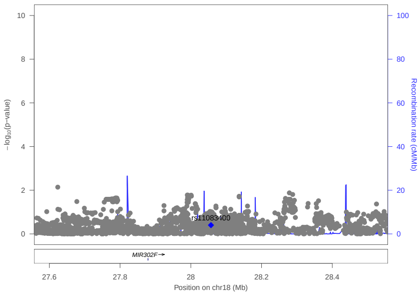

(J) *MIR302F*

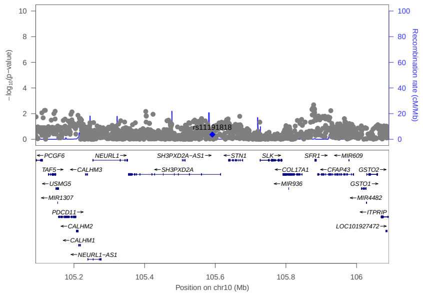

(K) *SH3PXD2A*

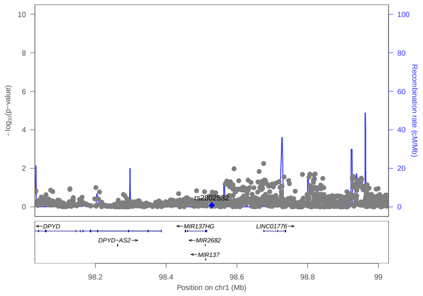

(L) *MIR137HG*

S14 Fig: Regional association plots to investigate if genetic overlap between CL/P & CP at any region of interest (as identified by PLACO in different pairwise analyses) is modified by sex. P-values from our SNP×Sex analyses are plotted. The blue diamond represents the most strongly associated SNP in the region of genetic overlap. For multi-ethnic analyses, there is no unique LD between SNPs and hence no color has been used to represent strength of LD.
