# Supplementary material for: Pleiotropy method reveals genetic overlap between orofacial clefts at multiple novel loci from GWAS of multi-ethnic trios
Source: PLoS Genet. 2021 Jul 9;17(7):e1009584. doi: 10.1371/journal.pgen.1009584 (PMC8270211; doi:10.1371/journal.pgen.1009584)
Supplement: S15 Fig — RR estimates are color annotated based on distance of SNPs from the index/lead SNP. LD-based color annotation is not used since these RR estimates are from multi-ethnic analyses and consequently, there is no unique LD between SNPs. Horizontal (vertical) error bar around each RR estimate corresponds to the 95% CI for the OFC subgroup represented on the x-axis (y-axis). The region depicting opposite genetic effects of SNPs for the 2 OFC subgroups is shaded in yellow. The number of SNPs in each quadrant is printed in the corresponding corner of the plot. While the 26 loci were identified from the gTDT analysis of CL/P at a suggestive threshold of 10−6, the SNPs plotted here are selected based on two criteria: (i) SNPs in ±500 Kb radius and in LD r2 > 0.2 with the index SNP (the most significant SNP in the locus); and (ii) SNPs with PLACO p-value <10−3 from the genetic overlap analysis of CL & CLP. Two loci, 6q22.31 and 19q13.11 (see S2 Table), are not depicted here since no SNP in these 2 loci show significance in PLACO analysis even at a liberal threshold of 10−3. These plots show the concordance of results for CL/P, and CL & CLP; i.e., the regions of genetic overlap identified by PLACO matches with the shared signals captured by the pooled analysis of CL and CLP subtypes. Additionally, there is some indication of genetically distinct etiology of subtypes CL and CLP at 1p22.1 (ABCA4, ARHGAP29) and 1q32.2 (IRF6), as depicted by the SNPs with opposite genetic effects. (PDF) [file pgen.1009584.s016.pdf]

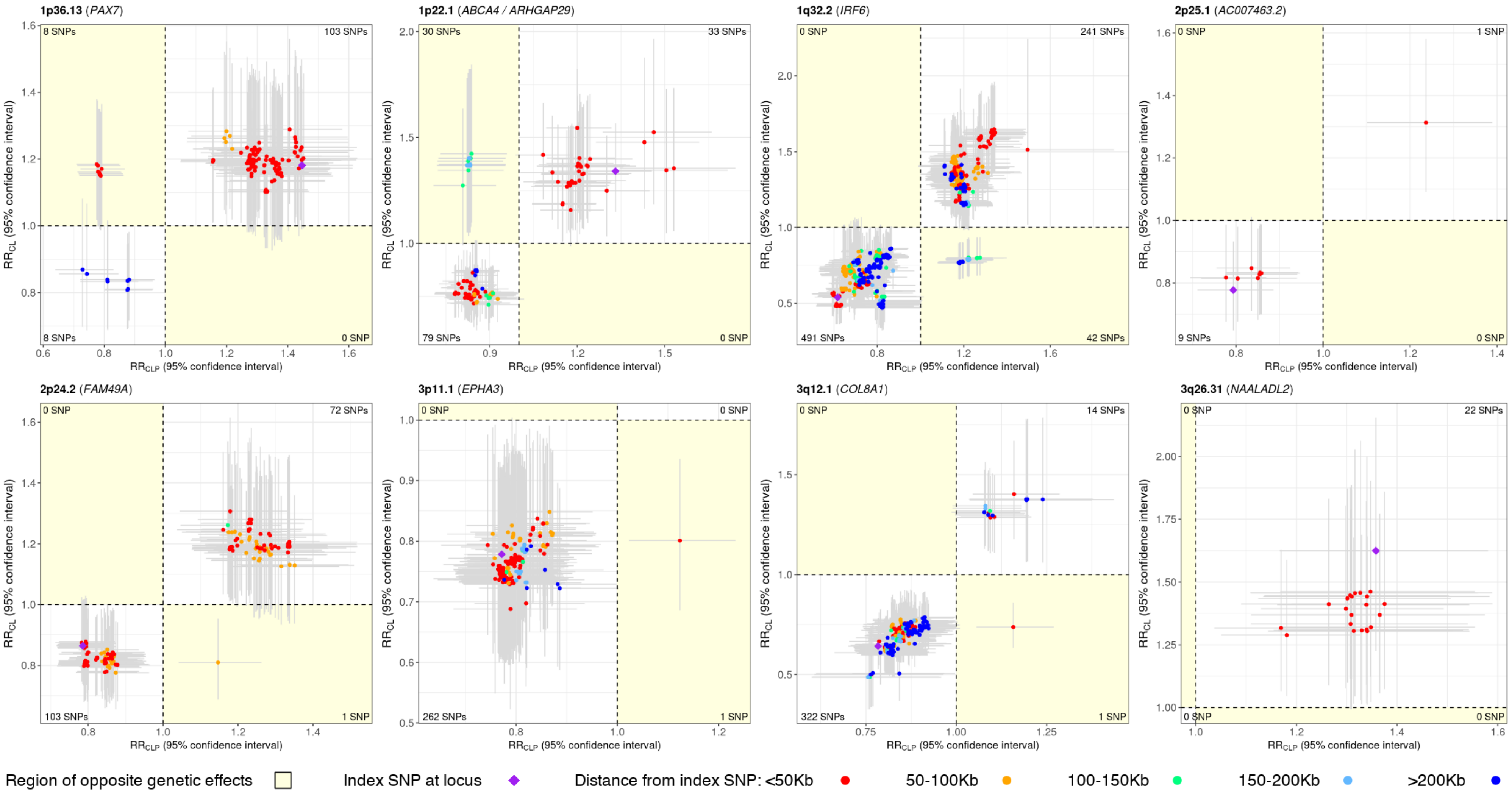


**S15 Fig: Scatter plot of relative risk (RR) estimates of CL and CLP, along with corresponding 95% confidence intervals (CIs), for variants in the 26 loci for CL/P.** RR estimates are color annotated based on distance of SNPs from the index/lead SNP. LD-based color annotation is not used since these RR estimates are from multi-ethnic analyses and consequently, there is no unique LD between SNPs. Horizontal (vertical) error bar around each RR estimate corresponds to the 95% CI for the OFC subgroup represented on the x-axis (y-axis). The region depicting opposite genetic effects of SNPs for the 2 OFC subgroups is shaded in yellow. The number of SNPs in each quadrant is printed in the corresponding corner of the plot. While the 26 loci were identified from the gTDT analysis of CL/P at a suggestive threshold of $10^{-6}$, the SNPs plotted here are selected based on two criteria: (i) SNPs in $\pm 500$ Kb radius and in LD $r^2 > 0.2$ with the index SNP (the most significant SNP in the locus); and (ii) SNPs with PLACO p-value $< 10^{-3}$ from the genetic overlap analysis of CL & CLP. Two loci, 6q22.31 and 19q13.11 (see S2 Table), are not depicted here since no SNP in these 2 loci show significance in PLACO analysis even at a liberal threshold of $10^{-3}$. These plots show the concordance of results for CL/P, and CL & CLP; i.e., the regions of genetic overlap identified by PLACO matches with the shared signals captured by the pooled analysis of CL and CLP subtypes. Additionally, there is some indication of genetically distinct etiology of subtypes CL and CLP at 1p22.1 (*ABCA4*, *ARHGAP29*) and 1q32.2 (*IRF6*), as depicted by the SNPs with opposite genetic effects. **(Figure continues over to next 2 pages)**

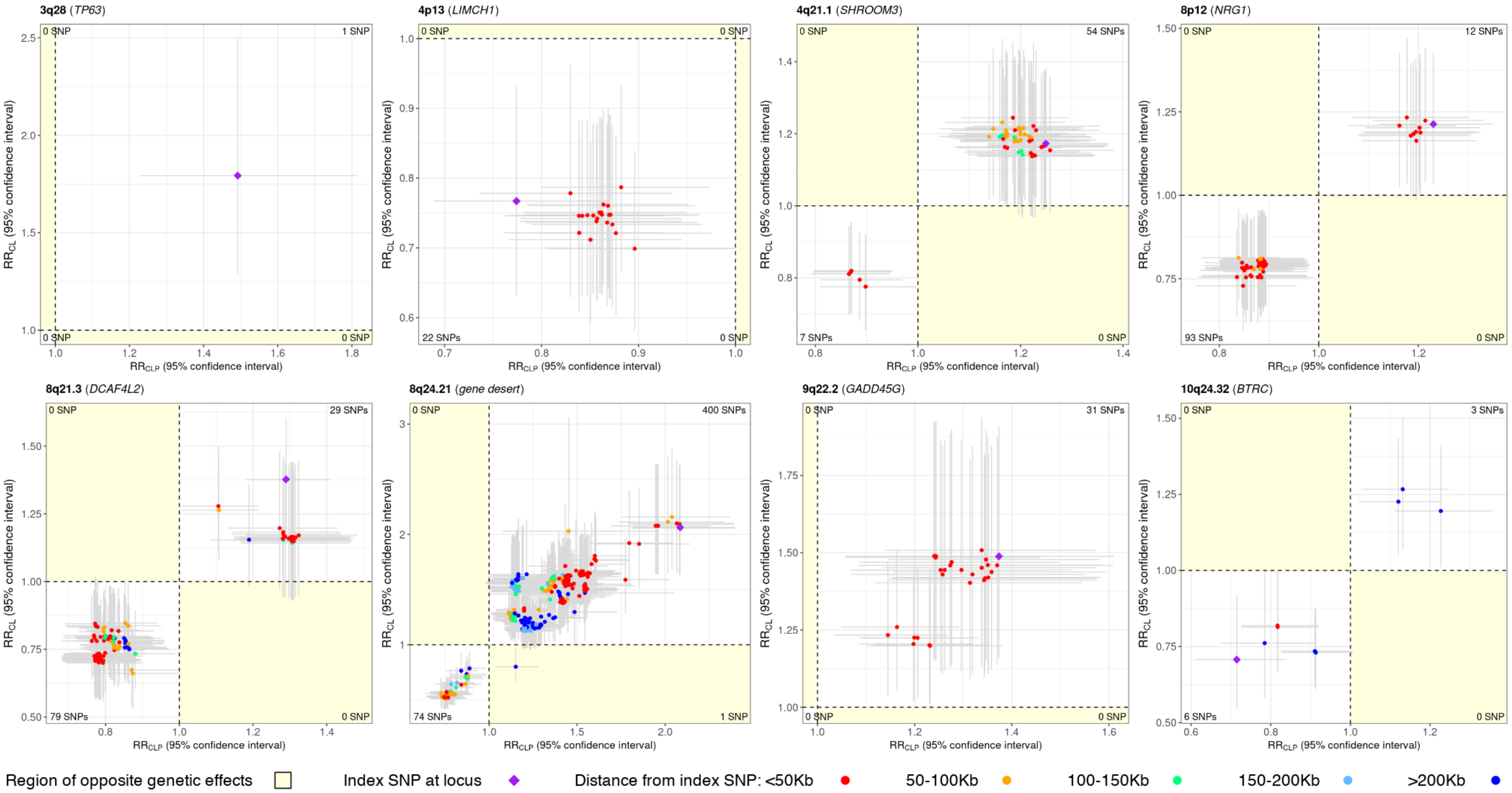


**S15 Fig: (Continued from previous page)** See previous page for caption.

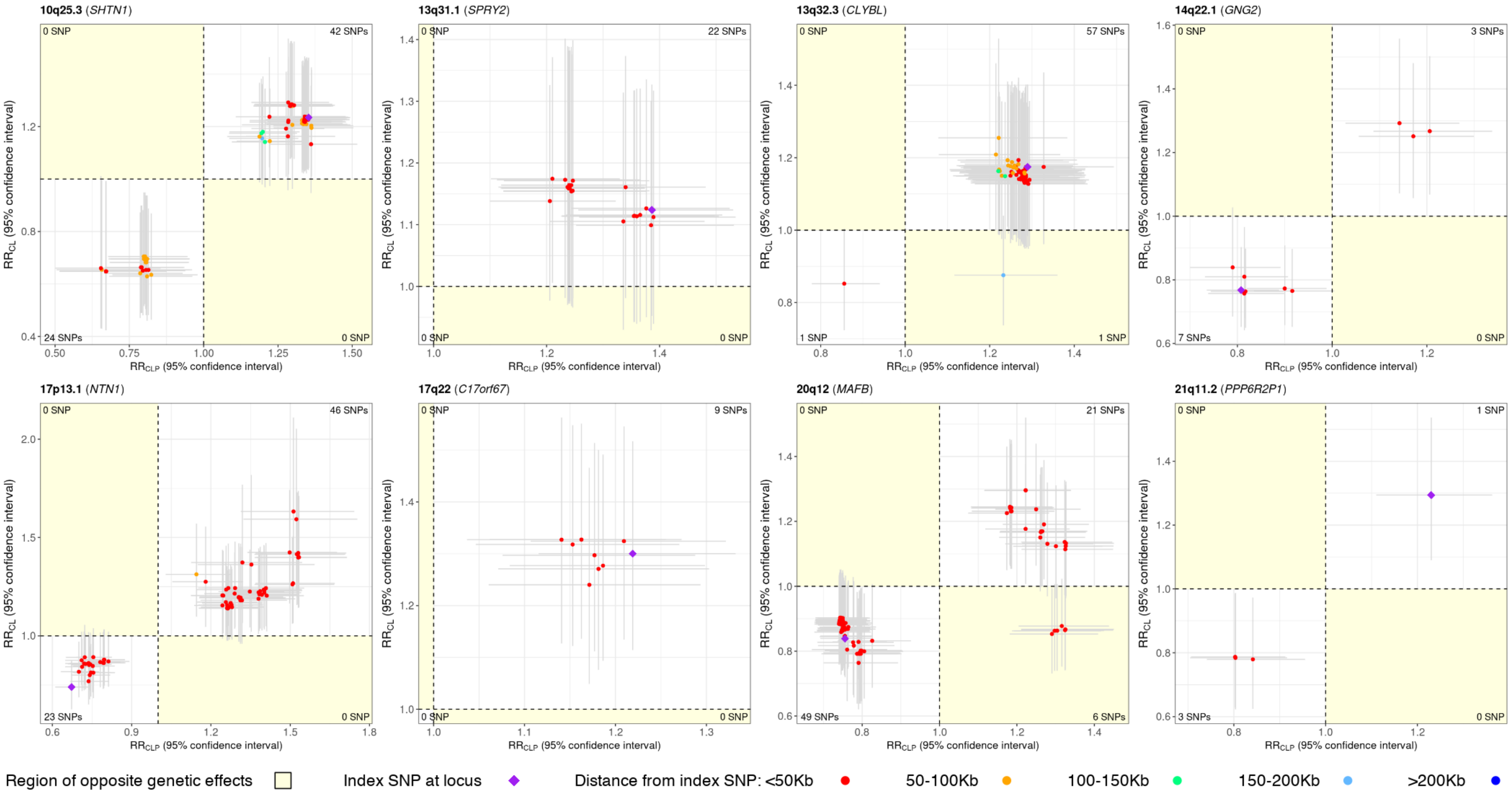


**S15 Fig: (Continued from previous page)** See previous page for caption.
