# Supplementary material for: Pleiotropy method reveals genetic overlap between orofacial clefts at multiple novel loci from GWAS of multi-ethnic trios
Source: PLoS Genet. 2021 Jul 9;17(7):e1009584. doi: 10.1371/journal.pgen.1009584 (PMC8270211; doi:10.1371/journal.pgen.1009584)
Supplement: S16 Fig — Index SNP here is the lead (most significant) SNP from the gTDT analysis of CL/P. SNPs with opposite genetic effects for the 2 OFC subtypes are colored in golden yellow while those with shared effects are in dark green. The directions of genetic effects are determined from the relative risk (RR) estimates for each subtype as provided by the gTDT method. RR estimates and the corresponding 95% confidence intervals for the SNPs above the dashed red horizontal line are portrayed in S15 Fig. Each of the loci here appear to have at least 2 distinct regions of genetic overlap. (PDF) [file pgen.1009584.s017.pdf]

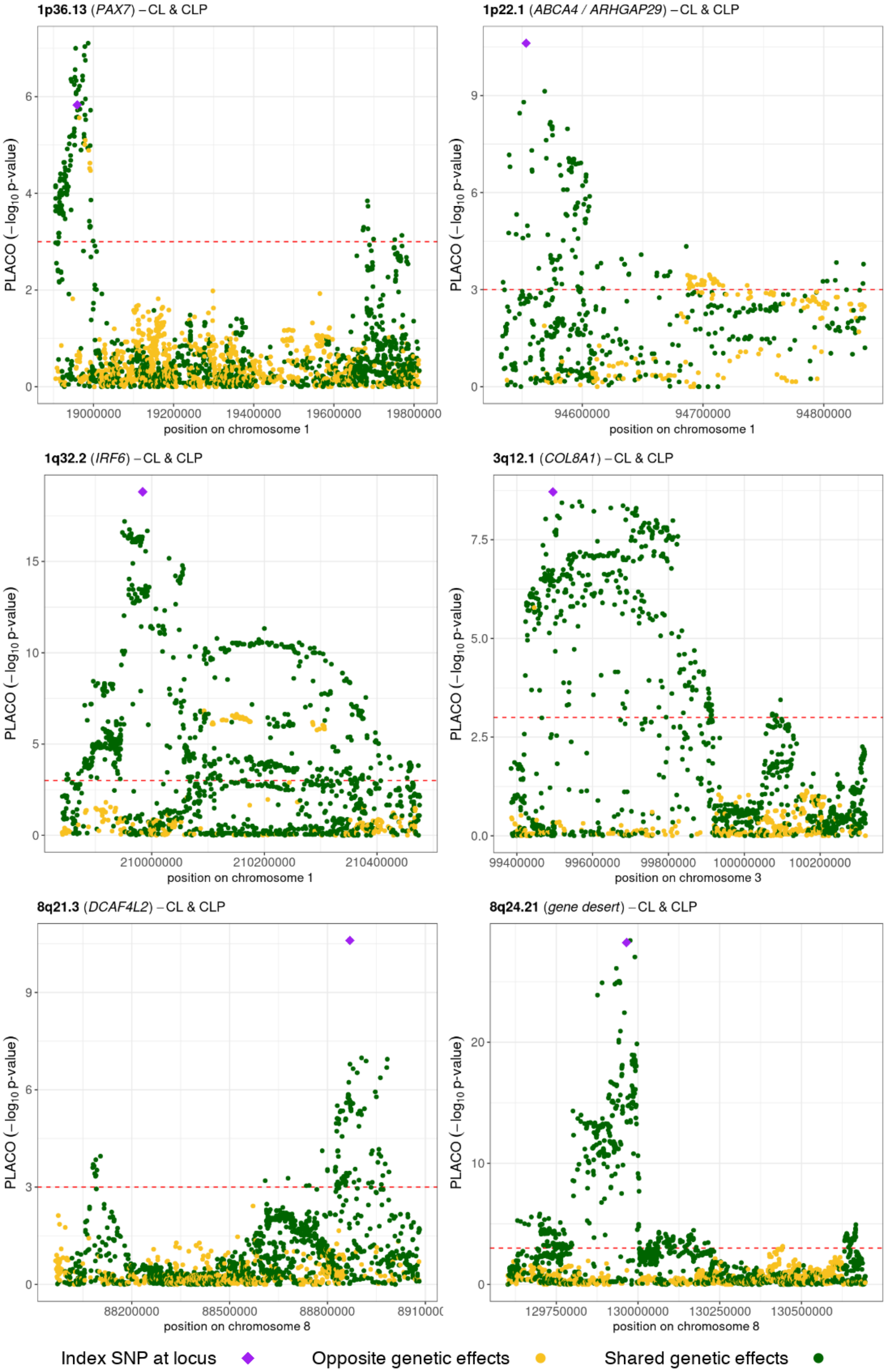

S16 Fig: Regional association plots of PLACO p-values from CL & CLP analysis, annotated by directions of effect sizes, for variants in some of the loci from the 26 loci for CL/P. Index SNP here is the lead (most significant) SNP from the gTDT analysis of CL/P. SNPs with opposite genetic effects for the 2 OFC subtypes are colored in golden yellow while those with shared effects are in dark green. The directions of genetic effects are determined from the relative risk (RR) estimates for each subtype as provided by the gTDT method. RR estimates and the corresponding 95% confidence intervals for the SNPs above the dashed red horizontal line are portrayed in S15 Fig. Each of the loci here appear to have at least 2 distinct regions of genetic overlap.
