# Supplementary material for: Pleiotropy method reveals genetic overlap between orofacial clefts at multiple novel loci from GWAS of multi-ethnic trios
Source: PLoS Genet. 2021 Jul 9;17(7):e1009584. doi: 10.1371/journal.pgen.1009584 (PMC8270211; doi:10.1371/journal.pgen.1009584)
Supplement: S17 Fig — LocusZoom plots focus on PLACO analysis of (A) CL & CLP, (E) CL & CLP annoted by directions of genetic effects, (F) CL/P & CP, (G) CL & CP, (H) CLP & CP, while (C) shows pooled method analysis of CL and CLP (i.e., gTDT analysis of subgroup CL/P). The blue diamond represents the most strongly associated SNP in the region showing evidence of genetic overlap. For multi-ethnic analyses, there is no unique LD between SNPs and hence no color has been used to represent strength of LD. Panel (B) shows relative risk (RR) estimates and their 95% confidence intervals (CIs) across OFC subtypes and racial/ethnic groups as obtained from the gTDT analyses. Panel (D) shows scatter plot of RR estimates of CL and CLP, along with corresponding 95% CIs, for the top several SNPs. SNPs represented here are in ±500 Kb radius and in LD r2 > 0.2 with the index SNP (the most significant SNP in the locus), and with PLACO p-value < 10−3 from the genetic overlap analysis of CL & CLP. RR estimates are color annotated based on distance of SNPs from the index/lead SNP. LD-based color annotation is not used since these RR estimates are from multi-ethnic analyses. Horizontal (vertical) error bar around each RR estimate corresponds to the 95% CI for CLP (CL). The region depicting opposite genetic effects of SNPs for the 2 OFC subtypes is shaded in yellow. The number of SNPs in each quadrant is printed in the corresponding corner of the plot. (PDF) [file pgen.1009584.s018.pdf]

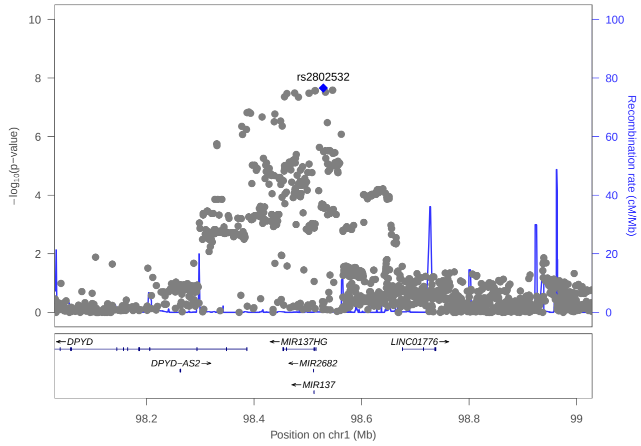


**(A)** CL & CLP (All ethnicities)

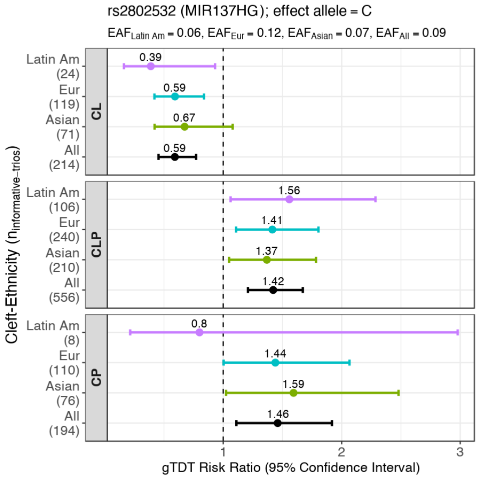


**(B)** RR estimates and 95% CIs

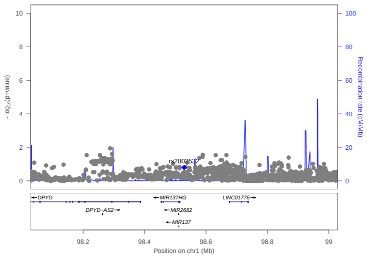

**(C)** Pooled CL + CLP (All ethnicities)

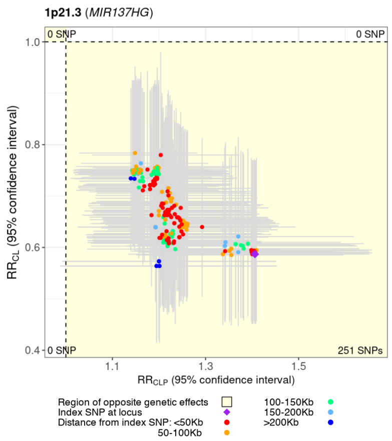


**(D)** Scatter plot of RR estimates – CL & CLP (All ethnicities)

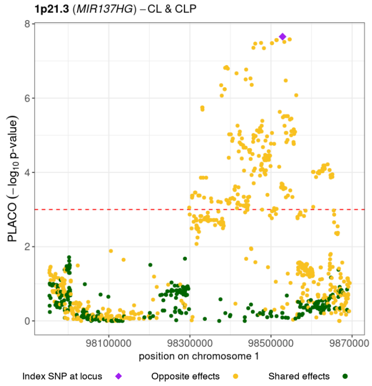


**(E)** CL & CLP (All ethnicities) showing effect directions

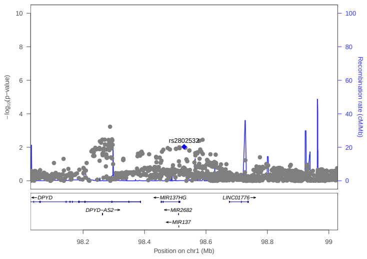

**(F)** CL/P & CP (All ethnicities)

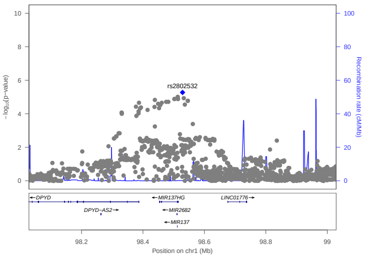

**(G)** CL & CP (All ethnicities)

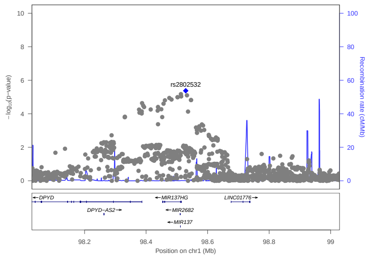

**(H)** CLP & CP (All ethnicities)

**S17 Fig: Regional association plots for 1p21.3 (*MIR137HG*) identified as a region of genetic overlap between CL & CLP.** LocusZoom plots focus on PLACO analysis of **(A)** CL & CLP, **(E)** CL & CLP annoted by directions of genetic effects, **(F)** CL/P & CP, **(G)** CL & CP, **(H)** CLP & CP, while **(C)** shows pooled method analysis of CL and CLP (i.e., gTDT analysis of subgroup CL/P). The blue diamond represents the most strongly associated SNP in the region showing evidence of genetic overlap. For multi-ethnic analyses, there is no unique LD between SNPs and hence no color has been used to represent strength of LD. Panel **(B)** shows relative risk (RR) estimates and their 95% confidence intervals (CIs) across OFC subtypes and racial/ethnic groups as obtained from the gTDT analyses. Panel **(D)** shows scatter plot of RR estimates of CL and CLP, along with corresponding 95% CIs, for the top several SNPs. SNPs represented here are in $\pm$500 Kb radius and in LD $r^2 > 0.2$ with the index SNP (the most significant SNP in the locus), and with PLACO p-value $< 10^{-3}$ from the genetic overlap analysis of CL & CLP. RR estimates are color annotated based on distance of SNPs from the index/lead SNP. LD-based color annotation is not used since these RR estimates are from multi-ethnic analyses. Horizontal (vertical) error bar around each RR estimate corresponds to the 95% CI for CLP (CL). The region depicting opposite genetic effects of SNPs for the 2 OFC subtypes is shaded in yellow. The number of SNPs in each quadrant is printed in the corresponding corner of the plot.
