# Supplementary material for: Pleiotropy method reveals genetic overlap between orofacial clefts at multiple novel loci from GWAS of multi-ethnic trios
Source: PLoS Genet. 2021 Jul 9;17(7):e1009584. doi: 10.1371/journal.pgen.1009584 (PMC8270211; doi:10.1371/journal.pgen.1009584)
Supplement: S18 Fig — Observed(−log10p-values) are plotted on the y-axis and Expected(−log10p-values) on the x-axis. Type I error performance of tests of simultaneous effect of a genetic variant on both outcomes is based on 9.99 million null variants with genetic effects that are either {RRCL/P = 1, RRCP = 1} or {RRCL/P = 1, RRCP = 1.15} or {RRCL/P = 1.15, RRCP = 1}. The gray shaded region represents a conservative 95% confidence interval for the expected distribution of p-values. P-values ≥ 10−12 are shown here. (PDF) [file pgen.1009584.s019.pdf]

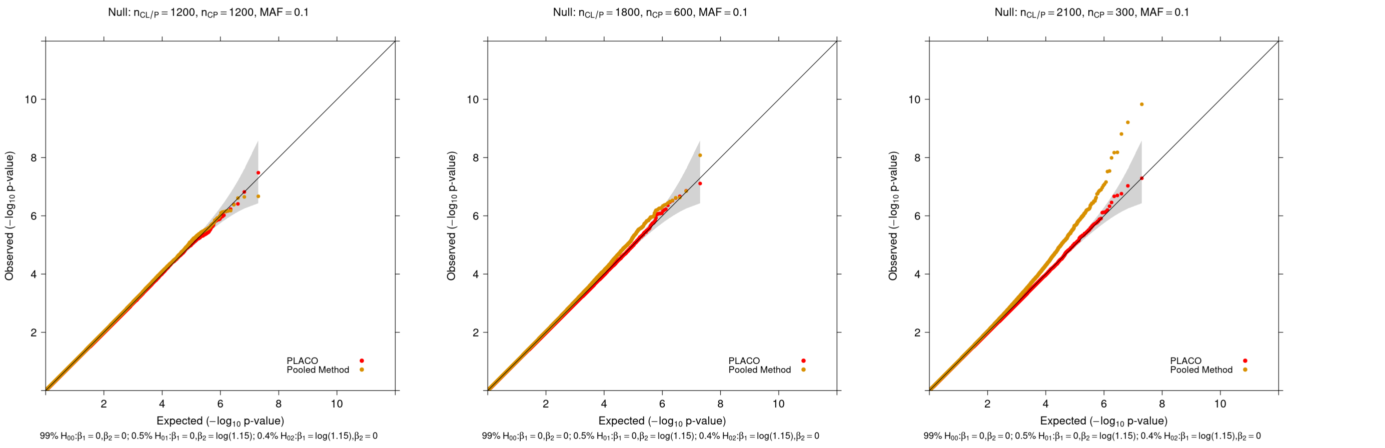

(A) MAF 10%; Balanced (1:1)

(B) MAF 10%; Unbalanced (3:1)

(C) MAF 10%; Heavily unbalanced (7:1)

S18 Fig: Scenario I: QQ plots for null data from two independent bi-ethnic case-parent trio studies of OFC subgroups assuming fixed genetic effects. Observed($-\log_{10}$p-values) are plotted on the y-axis and Expected($-\log_{10}$p-values) on the x-axis. Type I error performance of tests of simultaneous effect of a genetic variant on both outcomes is based on 9.99 million null variants with genetic effects that are either $\{RR_{CL/P}=1, RR_{CP}=1\}$ or $\{RR_{CL/P}=1, RR_{CP}=1.15\}$ or $\{RR_{CL/P}=1.15, RR_{CP}=1\}$. The gray shaded region represents a conservative 95% confidence interval for the expected distribution of p-values. P-values $\geq 10^{-12}$ are shown here.
