# Supplementary material for: Pleiotropy method reveals genetic overlap between orofacial clefts at multiple novel loci from GWAS of multi-ethnic trios
Source: PLoS Genet. 2021 Jul 9;17(7):e1009584. doi: 10.1371/journal.pgen.1009584 (PMC8270211; doi:10.1371/journal.pgen.1009584)
Supplement: S19 Fig — Observed(−log10p-values) are plotted on the y-axis and Expected(−log10p-values) on the x-axis. Type I error performance of tests of simultaneous effect of a genetic variant on both outcomes is based on 9.99 million null variants with genetic effects {log(RRCL/P) ∼ N(0, 0.12), RRCP = 1}. Values of RRCL/P ranged from 11.7 to 1.7. The gray shaded region represents a conservative 95% confidence interval for the expected distribution of p-values. P-values ≥ 10−12 are shown here. (PDF) [file pgen.1009584.s020.pdf]

Ray, D. et al. (2021) *Pleiotropy method reveals genetic overlap between orofacial clefts at multiple novel loci from GWAS of multi-ethnic trios.* PLoS Genetics, 17:e1009584, DOI: 10.1371/journal.pgen.1009584

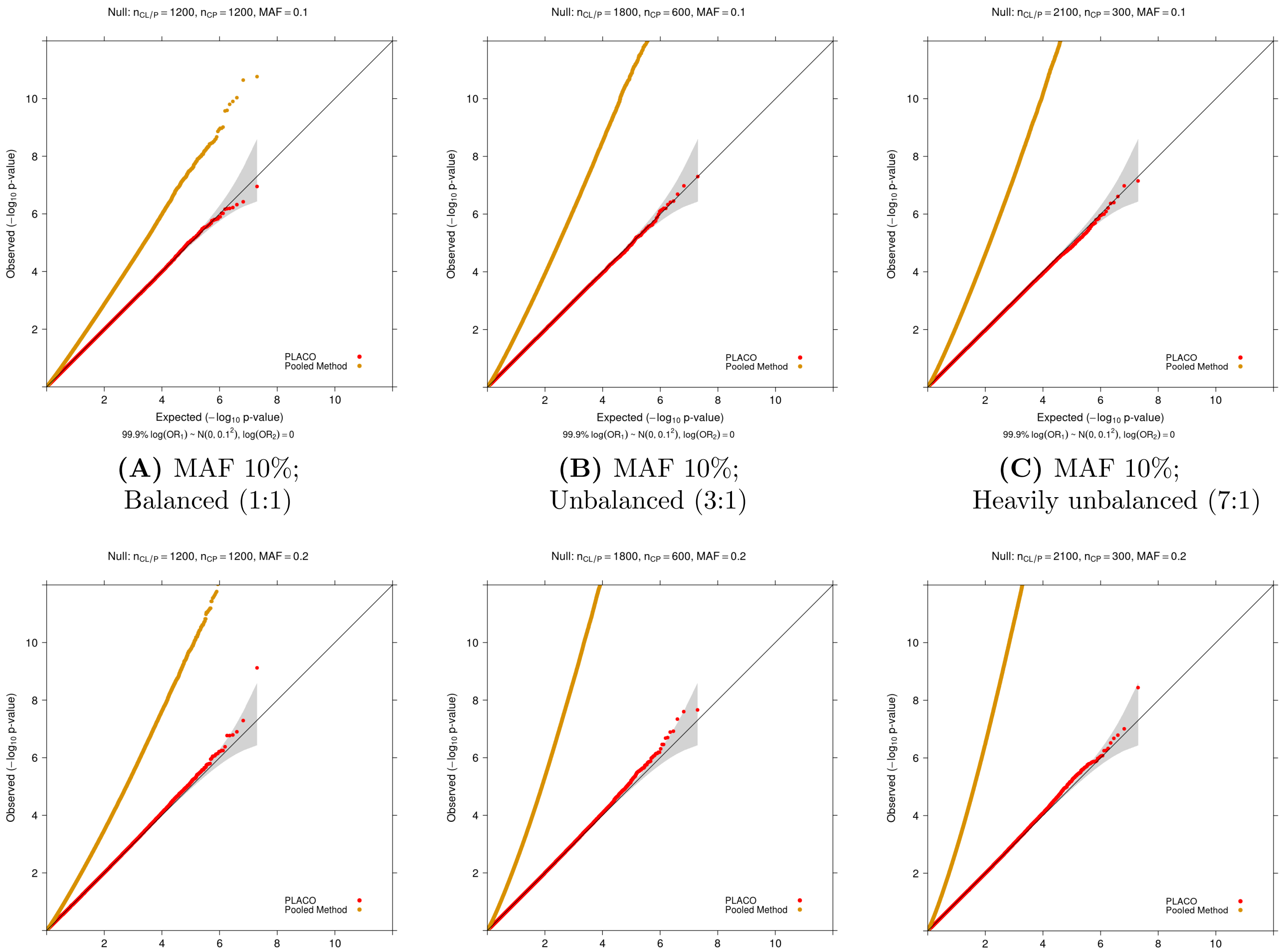

(A) MAF 10%; Balanced (1:1)

(B) MAF 10%; Unbalanced (3:1)

(C) MAF 10%; Heavily unbalanced (7:1)

(D) MAF 20%; Balanced (1:1)

(E) MAF 20%; Unbalanced (3:1)

(F) MAF 20%; Heavily unbalanced (7:1)

S19 Fig: Scenario II: QQ plots for null data from two independent bi-ethnic case-parent trio studies of OFC subgroups assuming fixed genetic effects for one trait and random for the other. Observed($-\log_{10}$p-values) are plotted on the y-axis and Expected($-\log_{10}$p-values) on the x-axis. Type I error performance of tests of simultaneous effect of a genetic variant on both outcomes is based on 9.99 million null variants with genetic effects $\{\log(\mathrm{RR}_{\mathrm{CL/P}}) \sim N(0, 0.1^2), \mathrm{RR}_{\mathrm{CP}} = 1\}$. Values of $\mathrm{RR}_{\mathrm{CL/P}}$ ranged from $\frac{1}{1.7}$ to 1.7. The gray shaded region represents a conservative 95% confidence interval for the expected distribution of p-values. P-values $\geq 10^{-12}$ are shown here.
