# Supplementary material for: Pleiotropy method reveals genetic overlap between orofacial clefts at multiple novel loci from GWAS of multi-ethnic trios
Source: PLoS Genet. 2021 Jul 9;17(7):e1009584. doi: 10.1371/journal.pgen.1009584 (PMC8270211; doi:10.1371/journal.pgen.1009584)
Supplement: S20 Fig — The first naive approach (‘Naive-1’) declares pleiotropic association when pCL/P < 5 × 10^−8^ and pCP < 10−5, while the second naive approach (‘Naive-2’) uses a more liberal criterion pCL/P < 5 × 10^−8^ and pCP < 10−3. Note, unlike PLACO, the pooled method lacks type I error control in most scenarios of sample size and/or MAF imbalance, and hence its power should be interpreted with caution. (PDF) [file pgen.1009584.s021.pdf]

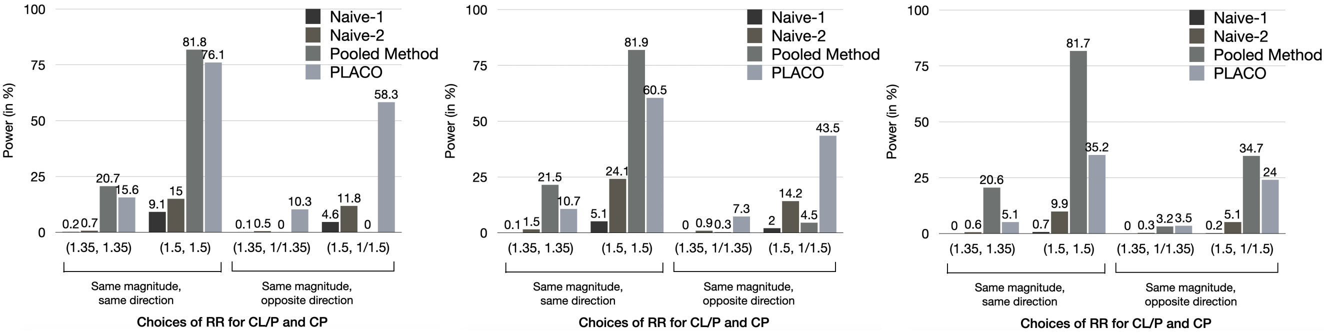

(A) MAF 10%; Balanced (1:1)

(B) MAF 10%; Unbalanced (3:1)

(C) MAF 10%; Heavily unbalanced (7:1)

S20 Fig: Power of PLACO, pooled method, and naive approaches at genome-wide significance level (5×10^{-8}) for varying genetic effects of the two independent bi-ethnic case-parent trio studies of OFC subgroups. The first naive approach ('Naive-1') declares pleiotropic association when $p_{\mathrm{CL/P}} < 5 \times 10^{-8}$ and $p_{\mathrm{CP}} < 10^{-5}$, while the second naive approach ('Naive-2') uses a more liberal criterion $p_{\mathrm{CL/P}} < 5 \times 10^{-8}$ and $p_{\mathrm{CP}} < 10^{-3}$. Note, unlike PLACO, the pooled method lacks type I error control in most scenarios of sample size and/or MAF imbalance, and hence its power should be interpreted with caution.
