# Supplementary material for: Pleiotropy method reveals genetic overlap between orofacial clefts at multiple novel loci from GWAS of multi-ethnic trios
Source: PLoS Genet. 2021 Jul 9;17(7):e1009584. doi: 10.1371/journal.pgen.1009584 (PMC8270211; doi:10.1371/journal.pgen.1009584)
Supplement: S21 Fig — The blue diamond represents the most strongly associated SNP in the region of genetic overlap. For multi-ethnic analyses, there is no unique LD between SNPs and hence no color has been used to represent strength of LD. (PDF) [file pgen.1009584.s022.pdf]

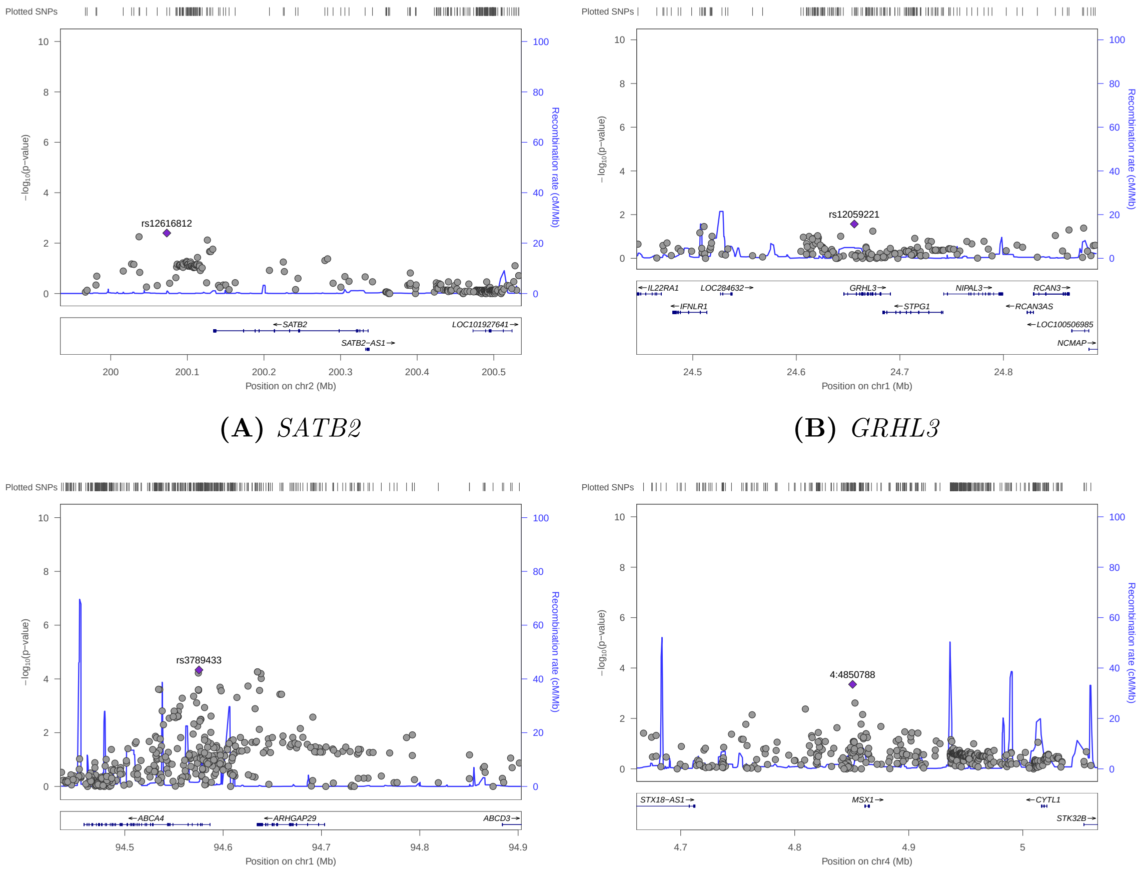

(A) *SATB2* **(B)** *GRHL3*

(C) *ARHGAP29* **(D)** *MSX1*

S21 Fig: Regional association plots to investigate if genetic overlap between CL/P & CP is identified by PLACO at some candidate regions based on past literature. The blue diamond represents the most strongly associated SNP in the region of genetic overlap. For multi-ethnic analyses, there is no unique LD between SNPs and hence no color has been used to represent strength of LD.
