# Supplementary material for: Pleiotropy method reveals genetic overlap between orofacial clefts at multiple novel loci from GWAS of multi-ethnic trios
Source: PLoS Genet. 2021 Jul 9;17(7):e1009584. doi: 10.1371/journal.pgen.1009584 (PMC8270211; doi:10.1371/journal.pgen.1009584)
Supplement: S1 Table — (PDF) [file pgen.1009584.s023.pdf]

S1 Table: Distribution of independent complete case-parent trios in the POFC and the GENEVA studies by racial/ethnic group and by cleft subtypes.

Study	Ethnicity	Sex*	CL	CLP	CP	All OFCs
POFC	All	All	263	1021	159	1443
		Female	104	365	95	564
		Male	159	656	64	879
	Asian	All	85	199	38	322
	European	All	89	314	93	496
	Latin American	All	89	508	28	625
GENEVA	All	All	431	1056	452	1939
		Female	186	348	254	788
		Male	245	708	198	1151
	Asian†	All	215	676	235	1126
	European	All	210	365	203	778
	Other	All	6	15	14	35

*Sex refers to the sex of the affected child in a trio.
†A handful of Malays from Singapore are present in the Asian racial/ethnic group of GENEVA. Recall, the GENEVA 'Asian' group consists of various Asian and southeast Asian ethnic groups (subjects recruited from People's Republic of China, Taiwan, South Korea, Singapore, and the Philippines).

Abbreviations: CL, cleft lip; CLP, cleft lip with palate; CP, cleft palate; OFCs, orofacial clefts
