# Supplementary material for: Pleiotropy method reveals genetic overlap between orofacial clefts at multiple novel loci from GWAS of multi-ethnic trios
Source: PLoS Genet. 2021 Jul 9;17(7):e1009584. doi: 10.1371/journal.pgen.1009584 (PMC8270211; doi:10.1371/journal.pgen.1009584)
Supplement: S2 Table — The analysis of CL/P is the same as pooled analysis of CL and CLP subtypes. Results from the genetic overlap analysis of CL & CLP using PLACO at these loci are also provided. Analyses are based on all trios from both POFC and GENEVA for a given cleft subtype. The “No. of trios” columns give the numbers of complete informative case-parent trios as used by gTDT. Note, the relevant 95% confidence intervals for the gTDT relative risk (RR) estimates here are provided in S15 Fig. CL & CLP PLACO p-value < 5 × 10^−8^ for a SNP indicates statistically significant association of the SNP with both CL and CLP at the genome-wide level. PLACO p-value < 10−6 is considered suggestive evidence of genetic overlap. (PDF) [file pgen.1009584.s024.pdf]

Ray, D. et al. (2021) *Pleiotropy method reveals genetic overlap between orofacial clefts at multiple novel loci from GWAS of multi-ethnic trios.* PLoS Genetics, 17:e1009584, DOI: 10.1371/journal.pgen.1009584

S2 Table: Association results for the most significant markers from the 26 loci for CL/P at a suggestive threshold of 10^{-6}. The analysis of CL/P is the same as pooled analysis of CL and CLP subtypes. Results from the genetic overlap analysis of CL & CLP using PLACO at these loci are also provided. Analyses are based on all trios from both POFC and GENEVA for a given cleft subtype. The "No. of trios" columns give the numbers of complete informative case-parent trios as used by gTDT. Note, the relevant 95% confidence intervals for the gTDT relative risk (RR) estimates here are provided in S15 Fig. CL & CLP PLACO p-value $< 5 \times 10^{-8}$ for a SNP indicates statistically significant association of the SNP with both CL and CLP at the genome-wide level. PLACO p-value $< 10^{-6}$ is considered suggestive evidence of genetic overlap.

					CL/P			CL			CLP			CL & CLP
Locus	Nearest gene	rsID	Position (hg19)	Effect allele	gTDT p-value	gTDT RR	No. of trios	gTDT p-value	gTDT RR	No. of trios	gTDT p-value	gTDT RR	No. of trios	PLACO p-value
1p36.13	*PAX7*	rs11308758	18959137	C	2.8×10^{-11}	1.37	1444	6.6×10^{-2}	1.18	391	3.0×10^{-11}	1.45	1053	1.5×10^{-6}
1p22.1	*ABCA4/ ARHGAP29*	rs560426	94553438	C	3.2×10^{-13}	1.33	2030	2.1×10^{-4}	1.34	508	3.6×10^{-10}	1.33	1522	2.4×10^{-11}
1q32.2	*IRF6*	rs11119346	209983900	T	2.0×10^{-21}	0.60	1187	8.1×10^{-8}	0.54	269	3.2×10^{-15}	0.62	918	1.5×10^{-19}
2p25.1	*AC007463.2*	rs35740468	8038388	T	7.7×10^{-7}	0.79	1366	6.6×10^{-3}	0.78	366	3.7×10^{-5}	0.79	1000	4.2×10^{-6}
2p24.2	*FAM49A*	rs4608519	16722908	G	8.6×10^{-9}	0.79	1874	7.1×10^{-2}	0.86	479	4.0×10^{-7}	0.79	1395	3.4×10^{-5}
3p11.1	*EPHA3*	rs11918555	89541934	T	6.9×10^{-9}	0.77	1636	3.8×10^{-3}	0.78	421	5.2×10^{-7}	0.77	1215	1.5×10^{-7}
3q12.1	*COL8A1*	rs793488	99495567	T	3.2×10^{-9}	0.75	1420	1.2×10^{-5}	0.64	353	1.7×10^{-5}	0.78	1067	1.9×10^{-9}
3q26.31	*NAALADL2*	rs144885328	174719127	G	3.4×10^{-7}	1.42	808	7.6×10^{-4}	1.62	190	7.8×10^{-5}	1.36	618	5.0×10^{-7}
3q28	*TP63*	rs74914009	189545021	G	1.7×10^{-7}	1.57	539	5.3×10^{-4}	1.79	144	6.1×10^{-5}	1.49	395	2.8×10^{-7}
4p13	*LIMCH1*	rs28609344	41640131	T	4.1×10^{-7}	0.77	1269	7.3×10^{-3}	0.77	337	1.8×10^{-5}	0.77	932	3.1×10^{-6}
4q21.1	*SHROOM3*	rs17002103	77512356	G	5.8×10^{-7}	1.23	1839	5.6×10^{-2}	1.17	460	3.1×10^{-6}	1.25	1379	4.4×10^{-5}
6q22.31	*CLVS2*	rs9490655	123417960	A	5.0×10^{-7}	0.81	1727	7.3×10^{-1}	1.03	416	8.7×10^{-1}	0.99	1311	8.6×10^{-1}
8p12	*NRG1*	rs1878918	32333570	C	6.2×10^{-7}	1.23	1869	2.0×10^{-2}	1.21	461	1.1×10^{-5}	1.23	1408	1.1×10^{-5}
8q21.3	*DCAF4L2*	rs12543318	88868340	C	3.3×10^{-12}	1.31	2032	4.5×10^{-5}	1.38	509	1.3×10^{-8}	1.29	1523	2.5×10^{-11}
8q24.21	8q24	rs17242358	129964873	A	1.4×10^{-33}	2.08	1028	5.4×10^{-10}	2.06	279	3.9×10^{-25}	2.09	749	5.8×10^{-29}
9q22.2	*GADD45G*	rs10908902	92224825	A	5.9×10^{-7}	1.41	732	1.7×10^{-3}	1.49	208	9.5×10^{-5}	1.37	524	1.5×10^{-6}
10q24.32	*BTRC*	rs11190939	103093456	A	6.8×10^{-7}	0.71	784	9.4×10^{-3}	0.71	203	2.4×10^{-5}	0.71	581	5.4×10^{-6}
10q25.3	*SHTN1*	rs10886042	118863209	A	9.9×10^{-11}	1.32	1745	1.5×10^{-2}	1.23	436	1.4×10^{-9}	1.35	1309	1.2×10^{-7}
13q31.1	*SPRY2*	rs1854110	80701485	C	3.0×10^{-10}	1.31	1717	1.7×10^{-1}	1.12	441	8.9×10^{-11}	1.39	1276	4.2×10^{-5}
13q32.3	*CLYBL*	rs9513637	100252331	T	5.4×10^{-7}	1.26	1522	7.4×10^{-2}	1.17	394	2.0×10^{-6}	1.29	1128	6.7×10^{-5}
14q22.1	*GNG2*	rs144433632	51856064	AAT	5.3×10^{-8}	0.80	1812	1.4×10^{-3}	0.77	464	9.2×10^{-6}	0.81	1348	2.1×10^{-7}
17p13.1	*NTN1*	rs12944377	8947708	C	1.6×10^{-18}	0.69	1809	3.5×10^{-4}	0.74	447	6.8×10^{-16}	0.67	1362	9.1×10^{-14}
17q22	*C17orf67*	rs227727	54776955	T	4.6×10^{-8}	1.24	1991	8.6×10^{-4}	1.30	501	1.2×10^{-5}	1.22	1490	1.4×10^{-7}
19q13.11	*RHPN2*	rs10417111	33504997	T	7.9×10^{-8}	0.66	654	5.2×10^{-1}	0.91	166	6.0×10^{-9}	0.60	488	9.7×10^{-3}
20q12	*MAFB*	rs11698990	39271008	G	7.2×10^{-11}	0.78	2042	2.4×10^{-2}	0.84	503	5.1×10^{-10}	0.76	1539	2.4×10^{-7}
21q11.2	*PPP6R2P1*	rs1297095	15380777	T	8.9×10^{-7}	1.25	1601	3.3×10^{-3}	1.29	416	7.3×10^{-5}	1.23	1185	2.6×10^{-6}

Abbreviations: Chr, chromosome; CL, cleft lip; CLP, cleft lip with palate; CL/P, cleft lip with or without palate; gTDT, genotypic transmission disequilibrium test; PLACO, pleiotropic analysis under composite null hypothesis; RR, relative risk (with respect to the reported effect allele)
